# Supplementary material for: Was the Last Bacterial Common Ancestor a Monoderm after All?
Source: Genes (Basel). 2022 Feb 18;13(2):376. doi: 10.3390/genes13020376 (PMC8871954; doi:10.3390/genes13020376)
Supplement: Supplementary file 1 [file genes-13-00376-s001.zip › genes-1558477-supplementary.pdf]

Supplementary Materials for

Was the last bacterial common ancestor a monoderm after all?

Raphaël R. Léonard [1,2], Eric Sauvage [1], Valérian Lupo [1,2], Amandine Perrin [3,4], Damien Sirjacobs [2], Paulette Charlier [1], Frédéric Kerff [1,*] and Denis Baurain [2,*]

[1] InBioS–Centre d'Ingénierie des Protéines, Université de Liège, Liège 4000, Belgium;
[2] InBioS–PhytoSYSTEMS, Unit of Eukaryotic Phylogenomics, Université de Liège, 4000 Liège, Belgium
[3] University Lille, CNRS, Centrale Lille, UMR 9189 CRIStAL, F-59000 Lille, France
[4] Hub de Bioinformatique et Biostatistique-Département Biologie Computationnelle, Institut Pasteur, Paris 75015, France

Running Title: A monoderm ancestor for the bacterial domain

* Correspondence: fkerff@uliege.be (F.K.); denis.baurain@uliege.be (D.B.)

Table S1: List of the 117 genes used for the phylogenomic tree of Figure S1. The genes are listed in their order of concatenation in the supermatrix. # species corresponds to the number of genomes (in the 101-species version ; see Figure S2) for which a given gene was present in the orthologous group (OG), and thus included here. # AA is the number of unambiguously aligned amino-acid positions used for each gene.

OG	# species	# AA	description
MCLdcw110100	100	246	L2
MCLdcw110104	101	175	S3
MCLdcw110105	100	138	S5
MCLdcw110107	100	126	L11
MCLdcw110109	98	111	hydrolase, TatD family
MCLdcw110112	99	109	L14
MCLdcw110114	100	76	S19
MCLdcw110116	101	451	translation initiation factor IF-2
MCLdcw110118	101	190	metalloendopeptidase, glycoprotease family
MCLdcw110124	100	116	S11
MCLdcw110125	96	246	cysteinyl-tRNA synthetase
MCLdcw110131	98	110	dimethyladenosine transferase
MCLdcw110132	100	200	DNA polymerase III, subunits gamma and tau
MCLdcw110139	96	383	GMP synthase
MCLdcw110140	97	124	tRNA pseudouridine synthase B
MCLdcw110159	98	124	S4
MCLdcw110162	98	109	tRNA dimethylallyltransferase
MCLdcw110169	94	292	Methionine adenosyltransferase
MCLdcw110172	98	372	aspartyl-tRNA synthetase
MCLdcw110178	100	188	DNA-directed RNA polymerase, alpha subunit
MCLdcw110179	98	446	CTP synthase
MCLdcw110188	99	118	S12
MCLdcw110189	100	153	L3
MCLdcw110190	99	92	L4/L1e
MCLdcw110192	99	189	S2

MCLdcw110195	101	126	L16
MCLdcw110198	100	276	UvrABC system protein C
MCLdcw110199	100	245	Peptide chain release factor 1
MCLdcw110202	101	69	L27
MCLdcw110204	100	120	L6
MCLdcw110205	100	87	L15
MCLdcw110206	99	90	L7/L12
MCLdcw110208	100	95	S9
MCLdcw110209	100	134	S7
MCLdcw110210	100	174	L5
MCLdcw110211	98	277	GTP-binding protein EngA
MCLdcw110214	100	155	DNA primase
MCLdcw110216	101	185	MraW
MCLdcw110217	100	108	L13
MCLdcw110218	100	79	L21
MCLdcw110219	101	265	GTP-binding protein Obg/CgtA
MCLdcw110222	100	564	Excinuclease ABC subunit B
MCLdcw110224	101	154	Ribosome-recycling factor
MCLdcw110225	100	95	S8
MCLdcw110226	101	116	S13
MCLdcw110228	97	129	Translation initiation factor IF-3
MCLdcw110230	100	85	L22
MCLdcw110233	100	128	SsrA-binding protein
MCLdcw110235	101	200	transcription elongation factor NusA
MCLdcw110239	101	112	L20
MCLdcw110242	100	194	L1
MCLdcw110243	100	161	tRNA-(guanine-N1)-methyltransferase
MCLdcw110244	99	79	S15
MCLdcw110246	100	40	L24
MCLdcw110247	100	90	L18
MCLdcw110248	101	258	preprotein translocase, SecY subunit

MCLdcw110249	98	76	L17
MCLdcw110253	93	234	UDP-N-acetylmuramoyl-L-alanyl-D-glutamate--2, 6-diaminopimelate ligase
MCLdcw110255	100	84	L19
MCLdcw110257	100	105	NusG antitermination factor
MCLdcw110258	100	232	Phenylalanyl-tRNA synthetase alpha chain
MCLdcw110259	101	60	S16
MCLdcw110260	100	97	S10
MCLdcw110265	98	91	L9
MCLdcw110269	93	229	Chorismate synthase
MCLdcw110270	100	73	S17
MCLdcw110272	98	183	Methionyl-tRNA formyltransferase
MCLdcw110273	101	183	uridylate kinase
MCLdcw110277	99	296	Holliday junction ATP-dependent DNA helicase ruvB
MCLdcw110287	98	323	lysyl-tRNA synthetase (class II)
MCLdcw110294	100	130	Guanylate kinase
MCLdcw110295	95	129	Phospho-N-acetylmuramoyl-pentapeptide-transferase
MCLdcw110297	100	129	riboflavin biosynthesis protein RibF
MCLdcw110298	87	85	tRNA threonylcarbamoyladenosine biosynthesis protein RimN
MCLdcw110306	99	304	Phenylalanyl-tRNA synthetase beta chain
MCLdcw110309	92	100	N-acetylglucosamine transferase
MCLdcw110313	96	108	pantetheine-phosphate adenylyltransferase
MCLdcw110314	96	170	glycerol-3-phosphate dehydrogenase
MCLdcw110317	92	56	L25/L23
MCLdcw110318	99	494	Polyribonucleotide nucleotidyltransferase
MCLdcw110321	95	134	Recombination protein recR
MCLdcw110327	93	56	L35
MCLdcw110332	92	238	Peptide chain release factor 2
MCLdcw110342	93	73	Holliday junction ATP-dependent DNA helicase ruvA
MCLdcw110345	87	29	S6
MCLdcw110349	94	502	transcription-repair coupling factor

MCLdcw110352	98	136	oxygen-independent coproporphyrinogen III oxidase
MCLdcw110353	91	134	DNA protecting protein DprA
MCLdcw110358	96	34	Uncharacterized protein family UPF0079, ATPase
MCLdcw110365	91	67	tRNA(Ile)-lysidine synthase
MCLdcw110373	87	338	ATP-dependent DNA helicase RecG
MCLdcw110380	90	126	pyrroline-5-carboxylate reductase
MCLdcw110383	92	213	DNA repair protein RecN
MCLdcw110388	92	52	Dephospho-CoA kinase
MCLdcw110394	90	116	6,7-dimethyl-8-ribityllumazine synthase
MCLdcw110405	97	370	Glutamyl-tRNA(Gln) amidotransferase subunit A
MCLdcw110408	93	48	iojap-like protein
MCLdcw110409	93	254	primosomal protein NÔÇÖ
MCLdcw110416	98	321	tRNA(Asn/Gln) amidotransferase subunit B
MCLdcw110420	98	73	L10
MCLdcw110425	93	66	nicotinate-nucleotide adenylyltransferase
MCLdcw110435	88	297	Argininosuccinate synthase
MCLdcw110444	90	136	Cytidylate kinase
MCLdcw110449	89	30	trigger factor
MCLdcw110457	91	105	Riboflavin synthase alpha chain
MCLdcw110466	89	222	S-adenosylmethionine: tRNA ribosyltransferase-isomerase
MCLdcw110494	88	79	4-diphosphocytidyl-2-C-methyl-D-erythritol kinase
MCLdcw110495	88	221	Imidazole glycerol phosphate synthase subunit hisF
MCLdcw110507	88	292	Porphobilinogen synthase
MCLdcw110513	89	338	chromosome segregation protein SMC
MCLdcw110524	87	96	Septum formation protein Maf
MCLdcw110525	90	72	crossover junction endodeoxyribonuclease RuvC
MCLdcw110556	93	224	1-deoxy-D-xylulose 5-phosphate reductoisomerase
MCLdcw110559	90	119	2C-methyl-D-erythritol 2,4-cyclodiphosphate synthase
MCLdcw110595	90	62	UPF0133 protein ybaB
MCLdcw110608	89	347	glutamate-1-semialdehyde-2,1-aminomutase
MCLdcw110617	92	194	fatty acid/phospholipid synthesis protein PlsX

Table S2: List of references used to determine the cell-wall architecture for the 85 representative organisms of Figure S1. To be used with Table S3.

1	Anderson, Iain, et al. "Genome sequence of the homoacetogenic bacterium Holophaga foetida type strain (TMBS4 T)." Standards in genomic sciences 6.2 (2012): 174-184.
2	Liesack, Werner, et al. "Holophaga foetida gen. nov., sp. nov., a new, homoacetogenic bacterium degrading methoxylated aromatic compounds." Archives of Microbiology 162.1 (1994): 85-90.
3	Pankratov, Timofei A., et al. "Substrate-induced growth and isolation of Acidobacteria from acidic Sphagnum peat." The ISME Journal 2.5 (2008): 551-560.
4	Kishimoto, Noriaki, Yoshimasa Kosako, and Tatsuo Tano. "Acidobacterium capsulatum gen. nov., sp. nov.: an acidophilic chemoorganotrophic bacterium containing menaquinone from acidic mineral environment." Current Microbiology 22.1 (1991): 1-7.
5	Carreto, Laura, et al. "Rubrobacter xylanophilus sp. nov., a new thermophilic species isolated from a thermally polluted effluent." International Journal of Systematic and Evolutionary Microbiology 46.2 (1996): 460-465.
6	Albuquerque, L., and M. S. da Costa. "The Family Rubrobacteraceae." The Prokaryotes (2014): 861-866.
7	Del Sol, Ricardo, et al. "Characterization of changes to the cell surface during the life cycle of Streptomyces coelicolor: atomic force microscopy of living cells." Journal of bacteriology 189.6 (2007): 2219-2225.
8	Bentley, S. D., et al. "SCP1, a 356 023 bp linear plasmid adapted to the ecology and developmental biology of its host, Streptomyces coelicolor A3 (2)." Molecular microbiology 51.6 (2004): 1615-1628.
9	Mohagheghi, A., et al. "Isolation and characterization of Acidothermus cellulolyticus gen. nov., sp. nov., a new genus of thermophilic, acidophilic, cellulolytic bacteria." International Journal of Systematic and Evolutionary Microbiology 36.3 (1986): 435-443.
10	Brüggemann, Holger, et al. "The complete genome sequence of Propionibacterium acnes, a commensal of human skin." Science 305.5684 (2004): 671-673.
11	Koskinen, Patrik, et al. "Complete genome sequence of Propionibacterium freudenreichii DSM 20271 T." Standards in genomic sciences 10.1 (2015): 1-6.
12	Del Rio, Tijana Glavina, et al. "Complete genome sequence of Chitinophaga pinensis type strain (UQM 2034 T)." Standards in genomic sciences 2.1 (2010): 87-95.
13	Alfredsson, Gudni A., et al. "Rhodothermus marinus, gen. nov., sp. nov., a thermophilic, halophilic bacterium from submarine hot springs in Iceland." Microbiology 134.2 (1988): 299-306.
14	Sharma, Shalley, et al. "Fulvivirga imtechensis sp. nov., a member of the phylum Bacteroidetes." International journal of systematic and evolutionary microbiology 62.Pt_9 (2012): 2213-2217.
15	Ueki, Atsuko, et al. "Paludibacter propionicigenes gen. nov., sp. nov., a novel strictly anaerobic, Gram-negative, propionate-producing bacterium isolated from plant residue in irrigated rice-field soil in Japan." International journal of systematic and evolutionary microbiology 56.1 (2006): 39-44.

16	Mori, Koji, et al. "Caldisericum exile gen. nov., sp. nov., an anaerobic, thermophilic, filamentous bacterium of a novel bacterial phylum, Caldiserica phyl. nov., originally called the candidate phylum OP5, and description of Caldisericaceae fam. nov., Caldisericales ord. nov. and Caldisericia classis nov." International journal of systematic and evolutionary microbiology 59.11 (2009): 2894-2898.
17	Matsumoto, Akira. "Structural characteristics of chlamydial bodies." Microbiology of chlamydia (1988): 21-45.
18	Frohlich, Kyla M., et al. "Membrane vesicle production by Chlamydia trachomatis as an adaptive response." Frontiers in cellular and infection microbiology 4 (2014): 73.
19	Everett, Karin DE, Robin M. Bush, and Arthur A. Andersen. "Emended description of the order Chlamydiales, proposal of Parachlamydiaceae fam. nov. and Simkaniaceae fam. nov., each containing one monotypic genus, revised taxonomy of the family Chlamydiaceae, including a new genus and five new species, and standards for the identification of organisms." International Journal of Systematic and Evolutionary Microbiology 49.2 (1999): 415-440.
20	Van Teeseling, Muriel CF, et al. "Anammox Planctomycetes have a peptidoglycan cell wall." Nature communications 6.1 (2015): 1-6.
21	Greub, G. "Parachlamydia acanthamoebae, an emerging agent of pneumonia." Clinical Microbiology and Infection 15.1 (2009): 18-28.
22	Michel, Rolf, et al. "Acanthamoeba from human nasal mucosa infected with an obligate intracellular parasite." European journal of protistology 30.1 (1994): 104-110.
23	Amann, Rudolf, et al. "Obligate intracellular bacterial parasites of acanthamoebae related to Chlamydia spp." Applied and environmental microbiology 63.1 (1997): 115-121.
24	Jacquier, Nicolas, et al. "Cell wall precursors are required to organize the chlamydial division septum." Nature communications 5.1 (2014): 1-11.
25	Everett, K. D., and Thomas P. Hatch. "Architecture of the cell envelope of Chlamydia psittaci 6BC." Journal of Bacteriology 177.4 (1995): 877-882.
26	Larsen, Helge. "On the culture and general physiology of the green sulfur bacteria." Journal of Bacteriology 64.2 (1952): 187-196.
27	Gibson, Jane, Norbert Pfennig, and John B. Waterbury. "Chloroherpeton thalassium gen. nov. et spec. nov., a non-filamentous, flexing and gliding green sulfur bacterium." Archives of microbiology 138.2 (1984): 96-101.
28	Pfennig, N. "Chlorobium phaeobacteroides nov. spec. and C. phaeovibrioides nov. spec., two new species of green sulfur bacteria." Archiv fur Mikrobiologie 63.3 (1968): 224-226.
29	Vogl, Kajetan, et al. "Chlorobium chlorochromatii sp. nov., a symbiotic green sulfur bacterium isolated from the phototrophic consortium “Chlorochromatium aggregatum”." Archives of microbiology 185.5 (2006): 363-372.
30	Reichenbach, Hans, and Jochen R. Golecki. "The fine structure of Herpetosiphon, and a note on the taxonomy of the genus." Archives of microbiology 102.1 (1975): 281-291.
31	Holt, J. G., and R. A. Lewin. "Herpetosiphon aurantiacus gen. et sp. n., a new filamentous gliding organism." Journal of bacteriology 95.6 (1968): 2407-2408.
32	Sekiguchi, Yuji, et al. "Anaerolinea thermophila gen. nov., sp. nov. and Caldilinea aerophila gen. nov., sp. nov., novel filamentous thermophiles that represent a previously uncultured lineage of the domain Bacteria at the subphylum level." International journal of systematic and evolutionary microbiology 53.6 (2003): 1843-1851.

33	Chang, Yun-Juan, et al. "Non-contiguous finished genome sequence and contextual data of the filamentous soil bacterium Ktedonobacter racemifer type strain (SOSP1-21 T)." Standards in genomic sciences 5.1 (2011): 97-111.
34	Cavaletti, Linda, et al. "New lineage of filamentous, spore-forming, gram-positive bacteria from soil." Applied and Environmental Microbiology 72.6 (2006): 4360-4369.
35	Wu, Dongying, et al. "Complete genome sequence of the aerobic CO-oxidizing thermophile Thermomicrobium roseum." PLoS One 4.1 (2009): e4207.
36	Merkel, G. J., D. R. Durham, and J. J. Perry. "The atypical cell wall composition of Thermomicrobium roseum." Canadian journal of microbiology 26.4 (1980): 556-559.
37	Rauschenbach, Ines, Priya Narasingarao, and Max M. Häggblom. "Desulfurispirillum indicum sp. nov., a selenate-and selenite-respiring bacterium isolated from an estuarine canal." International journal of systematic and evolutionary microbiology 61.3 (2011): 654-658.
38	Hoiczyk, Egbert, and Alfred Hansel. "Cyanobacterial cell walls: news from an unusual prokaryotic envelope." Journal of bacteriology 182.5 (2000): 1191-1199.
39	Schneider, Sabine, and Uwe J. Jürgens. "Cell wall and sheath constituents of the cyanobacterium Gloeobacter violaceus." Archives of microbiology 156.4 (1991): 312-318.
40	Myhr, Siri, and Terje Torsvik. "Denitrovibrio acetiphilus, a novel genus and species of dissimilatory nitrate-reducing bacterium isolated from an oil reservoir model column." International journal of systematic and evolutionary microbiology 50.4 (2000): 1611-1619.
41	Takai, Ken, et al. "Deferribacter desulfuricans sp. nov., a novel sulfur-, nitrate-and arsenate-reducing thermophile isolated from a deep-sea hydrothermal vent." international journal of systematic and evolutionary microbiology 53.3 (2003): 839-846.
42	Iino, Takao, et al. "Calditerrivibrio nitroreducens gen. nov., sp. nov., a thermophilic, nitrate-reducing bacterium isolated from a terrestrial hot spring in Japan." International journal of systematic and evolutionary microbiology 58.7 (2008): 1675-1679.
43	Fiala, Gerhard, et al. "Flexistipes sinusarabici, a novel genus and species of eubacteria occurring in the Atlantis II Deep brines of the Red Sea." Archives of Microbiology 154.2 (1990): 120-126.
44	Miroshnichenko, M. L., et al. "Oceanithermus profundus gen. nov., sp. nov., a thermophilic, microaerophilic, facultatively chemolithoheterotrophic bacterium from a deep-sea hydrothermal vent." International journal of systematic and evolutionary microbiology 53.3 (2003): 747-752.
45	Sako, Yoshihiko, et al. "Marinithermus hydrothermalis gen. nov., sp. nov., a strictly aerobic, thermophilic bacterium from a deep-sea hydrothermal vent chimney." International Journal of Systematic and Evolutionary Microbiology 53.1 (2003): 59-65.
46	Henne, Anke, et al. "The genome sequence of the extreme thermophile Thermus thermophilus." Nature biotechnology 22.5 (2004): 547-553.
47	Brooks, B. W., and R. G. E. Murray. "Nomenclature for “Micrococcus radiodurans” and other radiation-resistant cocci: Deinococcaceae fam. nov. and Deinococcus gen. nov., including five species." *International Journal of Systematic and Evolutionary Microbiology* 31.3 (1981): 353-360.
48	Geissinger, Oliver, et al. "The ultramicrobacterium “Elusimicrobium minutum” gen. nov., sp. nov., the first cultivated representative of the termite group 1 phylum." Applied and environmental microbiology 75.9 (2009): 2831-2840.
49	Jun, H. S., et al. "Fibrobacter succinogenes, a dominant fibrolytic ruminal bacterium: transition to the post genomic era." Asian-Australasian Journal of Animal Sciences 20.5 (2007): 802-810.
50	Driks, A. "Overview: development in bacteria: spore formation in Bacillus subtilis." Cellular and

	Molecular Life Sciences CMLS 59.3 (2002): 389-391.
51	Sekiguchi, Junichi, and Hiroki Yamamoto. "Cell wall structure of E. coli and B. subtilis, p 115–148." Escherichia coli and Bacillus subtilis: the frontiers of molecular microbiology revisited. Research Signpost, Kerala, India (2012).
52	Kay, D., and S. C. Warren. "Sporulation in Bacillus subtilis. Morphological changes." Biochemical Journal 109.5 (1968): 819-824.
53	Tocheva, Elitza I., et al. "Peptidoglycan transformations during B acillus subtilis sporulation." Molecular microbiology 88.4 (2013): 673-686.
54	Parshina, Sofiya N., et al. "Desulfotomaculum carboxydivorans sp. nov., a novel sulfate-reducing bacterium capable of growth at 100% CO." International Journal of Systematic and Evolutionary Microbiology 55.5 (2005): 2159-2165.
55	Campbell, L. LEON, and JOHN R. Postgate. "Classification of the spore-forming sulfate-reducing bacteria." Bacteriological reviews 29.3 (1965): 359-363.
56	He, Zengguo, et al. "Isolation and identification of a Paenibacillus polymyxa strain that coproduces a novel lantibiotic and polymyxin." Applied and Environmental Microbiology 73.1 (2007): 168-178.
57	Shelobolina, Evgenya S., et al. "Geobacter pickeringii sp. nov., Geobacter argillaceus sp. nov. and Pelosinus fermentans gen. nov., sp. nov., isolated from subsurface kaolin lenses." International Journal of Systematic and Evolutionary Microbiology 57.1 (2007): 126-135.
58	Tang, Kuo-Hsiang, Hai Yue, and Robert E. Blankenship. "Energy metabolism of Heliobacterium modesticaldum during phototrophic and chemotrophic growth." BMC microbiology 10.1 (2010): 1-14.
59	Sikorski, Johannes, et al. "Complete genome sequence of Acetohalobium arabaticum type strain (Z-7288 T)." Standards in genomic sciences 3.1 (2010): 57-65.
60	Tocheva, Elitza I., et al. "Peptidoglycan remodeling and conversion of an inner membrane into an outer membrane during sporulation." Cell 146.5 (2011): 799-812.
61	Mori, Koji, et al. "A novel lineage of sulfate-reducing microorganisms: Thermodesulfobiaceae fam. nov., Thermodesulfobium narugense, gen. nov., sp. nov., a new thermophilic isolate from a hot spring." Extremophiles 7.4 (2003): 283-290.
62	MURRAY, WILLIAM D., and A. W. Khan. "Clostridium saccharolyticum sp. nov., a saccharolytic species from sewage sludge." International Journal of Systematic and Evolutionary Microbiology 32.1 (1982): 132-135.
63	Van Gylswyk, N. O., E. JANE MORRIS, and H. J. Els. "Sporulation and cell wall structure of Clostridium polysaccharolyticum comb. nov.(formerly Fusobacterium polysaccharolyticum)." Microbiology 121.2 (1980): 491-493.
64	Umeda, Akiko, Y. U. J. I. Ueki, and K. A. Z. U. N. O. B. U. Amako. "Structure of the Staphylococcus aureus cell wall determined by the freeze-substitution method." Journal of bacteriology 169.6 (1987): 2482-2487.
65	Kosowska, Klaudia, et al. "The Clostridium ramosum IgA proteinase represents a novel type of metalloendopeptidase." Journal of Biological Chemistry 277.14 (2002): 11987-11994.
66	Zhang, Hui, et al. "Gemmatimonas aurantiaca gen. nov., sp. nov., a Gram-negative, aerobic, polyphosphate-accumulating micro-organism, the first cultured representative of the new bacterial phylum Gemmatimonadetes phyl. nov." International journal of systematic and evolutionary microbiology 53.4 (2003): 1155-1163.

67	Kadnikov, Vitaly V., et al. "Characterization of Melioribacter roseus gen. nov., sp. nov., a novel facultatively anaerobic thermophilic cellulolytic bacterium from the class Ignavibacteria, and a proposal of a novel bacterial phylum Ignavibacteriae." (2013).
68	Iino, Takao, et al. "Ignavibacterium album gen. nov., sp. nov., a moderately thermophilic anaerobic bacterium isolated from microbial mats at a terrestrial hot spring and proposal of Ignavibacteria classis nov., for a novel lineage at the periphery of green sulfur bacteria." International journal of systematic and evolutionary microbiology 60.6 (2010): 1376-1382.
69	Cho, Jang-Cheon, et al. "Lentisphaera araneosa gen. nov., sp. nov, a transparent exopolymer producing marine bacterium, and the description of a novel bacterial phylum, Lentisphaerae." Environmental Microbiology 6.6 (2004): 611-621
70	Henry, E. A., et al. "Characterization of a new thermophilic sulfate-reducing bacterium." Archives of Microbiology 161.1 (1994): 62-69.
71	Hippe, Hans. "Leptospirillum gen. nov.(ex Markosyan 1972), nom. rev., including Leptospirillum ferrooxidans sp. nov.(ex Markosyan 1972), nom. rev. and Leptospirillum thermoferrooxidans sp. nov.(Golovacheva et al. 1992)." International journal of systematic and evolutionary microbiology 50.2 (2000): 501-503.
72	Van Teeseling, Muriel CF, et al. "Anammox Planctomycetes have a peptidoglycan cell wall." Nature communications 6.1 (2015): 1-6.
73	Nevin, Kelly P., et al. "Geobacter bemidjiensis sp. nov. and Geobacter psychrophilus sp. nov., two novel Fe (III)-reducing subsurface isolates." International Journal of Systematic and Evolutionary Microbiology 55.4 (2005): 1667-1674.
74	Bazylinski, Dennis A., et al. "Magnetococcus marinus gen. nov., sp. nov., a marine, magnetotactic bacterium that represents a novel lineage (Magnetococcaceae fam. nov., Magnetococcales ord. nov.) at the base of the Alphaproteobacteria." International journal of systematic and evolutionary microbiology 63.Pt_3 (2013): 801-808.
75	Bryantseva, Irina, et al. "Thiorhodospira sibirica gen. nov., sp. nov., a new alkaliphilic purple sulfur bacterium from a Siberian soda lake." International Journal of Systematic and Evolutionary Microbiology 49.2 (1999): 697-703.
76	Matias, Valério RF, et al. "Cryo-transmission electron microscopy of frozen-hydrated sections of Escherichia coli and Pseudomonas aeruginosa." Journal of bacteriology 185.20 (2003): 6112-6118.
77	Keck, Matthias, et al. "Unusual outer membrane lipid composition of the gram-negative, lipopolysaccharide-lacking myxobacterium Sorangium cellulosum So ce56." Journal of Biological Chemistry 286.15 (2011): 12850-12859.
78	Koops, H. P., and G. Stehr. "Classification of eight new species of ammonia-oxidizing bacteria: Nitrosomonas communis sp. nov., Nitrosomonas ureae sp. nov., Nitrosomonas aestuarii sp. nov., Nitrosomonas marina sp. nov., Nitrosomonas nitrosa sp. nov., Nitrosomonas eutropha sp. nov., Nitrosomonas oligotropha sp. nov. and Nitrosomonas halophila sp. nov." Microbiology 137.7 (1991): 1689-1699.
79	Pati, Amrita, et al. "Complete genome sequence of Arcobacter nitrofigilis type strain (CI T)." Standards in Genomic Sciences 2.3 (2010): 300-308.
80	Curtis, Patrick D., and Yves V. Brun. "Getting in the loop: regulation of development in Caulobacter crescentus." Microbiology and Molecular Biology Reviews 74.1 (2010): 13-41.
81	Hill, Darryl J., et al. "Cellular and molecular biology of Neisseria meningitidis colonization and invasive disease." Clinical science 118.9 (2010): 547-564.
82	Mobley, Harry LT, George L. Mendz, and Stuart L. Hazell. "Helicobacter pylori: physiology and

	genetics." (2001).
83	Huntemann, Marcel, et al. "Genome sequence of the phylogenetically isolated spirochete Leptonema illini type strain (3055 T)." Standards in genomic sciences 8.2 (2013): 177-187.
84	Johnson, Russell C. “Leptospira.” Medical Microbiology, edited by Samuel Baron, 4th ed., University of Texas Medical Branch at Galveston, 1996.
85	Stackebrandt, Erko, et al. "Genome sequence of the free-living aerobic spirochete Turneriella parva type strain (HT), and emendation of the species Turneriella parva." Standards in genomic sciences 8.2 (2013): 228-238.
86	Evangelista, Karen V., and Jenifer Coburn. "Leptospira as an emerging pathogen: a review of its biology, pathogenesis and host immune responses." Future microbiology 5.9 (2010): 1413-1425.
87	Razin, Shmuel. "Isolation and characterization of mycoplasma membranes." The mycoplasmas 1 (1979): 213-229.
88	Moussard, H., et al. "Thermodesulfatator indicus gen. nov., sp. nov., a novel thermophilic chemolithoautotrophic sulfate-reducing bacterium isolated from the Central Indian Ridge." International Journal of Systematic and Evolutionary Microbiology 54.1 (2004): 227-233.
89	Hamilton-Brehm, Scott D., et al. "Thermodesulfobacterium geofontis sp. nov., a hyperthermophilic, sulfate-reducing bacterium isolated from Obsidian Pool, Yellowstone National Park." Extremophiles 17.2 (2013): 251-263.
90	Kant, Ravi, et al. "Genome sequence of “Pedosphaera parvula” Ellin514, an aerobic verrucomicrobial isolate from pasture soil." (2011): 2900-2901.
91	Schlesner, Heinz. The Prokaryotes: An Evolving Electronic Resource for the Microbiological Community: The Genus Verrucomicrobium. 2004. Springer-Verlag New York, LLC.
92	Chin, Kuk-Jeong, Werner Liesack, and Peter H. Janssen. "Opitutus terrae gen. nov., sp. nov., to accommodate novel strains of the division'Verrucomicrobia'isolated from rice paddy soil." International journal of systematic and evolutionary microbiology 51.6 (2001): 1965-1968.
93	Miroshnichenko, Margarita L., et al. "Caldithrix abyssi gen. nov., sp. nov., a nitrate-reducing, thermophilic, anaerobic bacterium isolated from a Mid-Atlantic Ridge hydrothermal vent, represents a novel bacterial lineage." International journal of systematic and evolutionary microbiology 53.1 (2003): 323-329.
94	Botero, Lina M., et al. "Thermobaculum terrenum gen. nov., sp. nov.: a non-phototrophic gram-positive thermophile representing an environmental clone group related to the Chloroflexi (green non-sulfur bacteria) and Thermomicrobia." Archives of microbiology 181.4 (2004): 269-277.

Table S3: Details of the data given to BayesTraits for the ancestral trait reconstruction. Trailing numbers after organism names are NCBI Taxonomy identifiers. In the reference column, the reference corresponding to the number can be found in Table S2. Peptidoglycan (PG): 0 = no PG, 1= thin PG, 2 = thick PG; membrane (MBN full): 0 = monoderm, 1 = diderm without lipopolysaccharides (LPS), 2 = diderm with LPS; spore: 0 = no spore, 1 = endospore, 2 = exospore, 3 = myxospore; membrane (MBN simple): 0 = monoderm, 1 = diderm.

organism	phylum	PG	MBN (full)	spore	MBN (simple)	ref	taxonomic level of the information
Acetohalobium_arabaticum_574087	Firmicutes	1	2	1	1	59;60	species

Acholeplasma_laidlawii_441768	Tenericutes	0	0	0	0	87	class
Acidobacterium_capsulatum_240015	Acidobacteria	1	12	0	1	3;4	species
Acidothermus_cellulolyticus_351607	Actinobacteria	2	0	0	0	9	species
Arcobacter_nitrofigilis_572480	Proteobacteria	1	12	0	1	79	species
Bacillus_subtilis_1147161	Firmicutes	2	0	1	0	50-53	species
Caldilinea_aerophila_926550	Chloroflexi	1	12	0	1	32	species
Caldisericum_exile_511051	Caldiserica	1	12	0	1	16	species
Calditerrivibrio_nitroreducens_768670	Deferribacteres	1	12	0	1	42	species
Caldithrix_abyssi_880073	undef	1	12	0	1	93	species
Caulobacter_crescentus_190650	Proteobacteria	1	12	0	1	80	species
Chitinophaga_pinensis_485918	Bacteroidetes	1	12	3	1	12	species
Chlamydia_psittaci_1238235	Chlamydiae	1	2	0	1	17-20;25	species
Chlamydophila_pneumoniae_115711	Chlamydiae	1	2	0	1	17-20	phylum
Chlorobaculum_parvum_517417	Chlorobi	1	12	0	1	26	phylum
Chlorobium_chlorochromatii_340177	Chlorobi	1	12	0	1	26;29	species
Chlorobium_phaeovibrioides_290318	Chlorobi	1	12	0	1	26;28	species
Chloroherpeton_thalassium_517418	Chlorobi	1	12	0	1	26;27	species
Clostridium_ramosum_445974	Firmicutes	2	0	1	0	65	species
Clostridium_saccharolyticum_610130	Firmicutes	2	0	1	0	62;63	species
Deferribacter_desulfuricans_639282	Deferribacteres	1	12	0	1	41	species
Deinococcus_deserti_546414	Deinococcus-Thermus	2	2	0	1	47	species
Denitrovibrio_acetiphilus_522772	Deferribacteres	1	12	0	1	40	species
Desulfotomaculum_carboxydivorans_868595	Firmicutes	2	0	1	0	54;55	species
Desulfurispirillum_indicum_653733	Chrysiogenetes	1	12	0	1	37	species
Elusimicrobium_minutum_445932	Elusimicrobia	1	12	0	1	48	species
Escherichia_coli_574521	Proteobacteria	1	2	0	1	76	species
Fibrobacter_succinogenes_59374	Fibrobacteres	1	12	0	1	49	species
Flexistipes_sinusarabici_717231	Deferribacteres	1	12	0	1	43	species

Fulvivirga_imtechensis_1237149	Bacteroidetes	1	12	0	1	14	species
Gemmatimonas_aurantiaca_379066	Gemmatimonadetes	1	12	0	1	66	species
Geobacter_bemidjiensis_404380	Proteobacteria	1	12	0	1	73	species
Gloeobacter_violaceus_251221	Cyanobacteria	1	2	0	1	38;39	species
Helicobacter_pylori_85963	Proteobacteria	1	12	0	1	82	species
Heliobacterium_modesticaldum_498761	Firmicutes	2	0	1	0	58	species
Herpetosiphon_aurantiacus_316274	Chloroflexi	2	12	0	1	30;31	species
Holophaga_foetida_903818	Acidobacteria	1	12	0	1	1;2	species
Ignavibacterium_album_945713	Ignavibacteriae	1	12	0	1	68	species
Ktedonobacter_racemifer_485913	Chloroflexi	2	0	2	0	33;34	species
Lentisphaera_araneosa_313628	Lentisphaerae	1	12	0	1	69	species
Leptonema_illini_929563	Spirochaetes	1	12	0	1	83	species
Leptospira_interrogans_189518	Spirochaetes	1	2	0	1	84	species
Leptospira_meyeri_1218585	Spirochaetes	1	2	0	1	86	species
Leptospirillum_ferrooxidans_1162668	Nitrospirae	1	12	0	1	71	species
Magnetococcus_marinus_156889	Proteobacteria	1	12	0	1	74	species
Marinithermus_hydrothermalis_869210	Deinococcus-Thermus	1	12	0	1	45	species
Melioribacter_roseus_1191523	Ignavibacteriae	1	12	0	1	67	species
Mycoplasma_penetrans_272633	Tenericutes	0	0	0	0	87	class
Neisseria_meningitidis_662598	Proteobacteria	1	2	0	1	81	species
Nitrosomonas_sp._153948	Proteobacteria	1	12	0	1	78	species
Oceanithermus_profundus_670487	Deinococcus-Thermus	1	12	0	1	44	species
Opitutaceae_bacterium_278956	Verrucomicrobia	1	12	0	1	92	other species
Opitutus_terrae_452637	Verrucomicrobia	1	12	0	1	92	species
Oscillatoriales_cyanobacterium_864702	Cyanobacteria	2	2	0	1	38	phylum
Paenibacillus_polymyxa_886882	Firmicutes	2	0	0	0	56	species
Paludibacter_propionicigenes_694427	Bacteroidetes	1	12	0	1	15	species
Parachlamydia_acanthamoebae_765952	Chlamydiae	12	2	0	1	17-23	species
Pedosphaera_parvula_320771	Verrucomicrobia	1	12	0	1	90	species

Pelosinus_fermentans_1122947	Firmicutes	1	12	1	1	57	species
Planctomyces_brasiliensis_756272	Planctomycetes	1	12	0	1	72	phylum
Planctomyces_maris_344747	Planctomycetes	1	12	0	1	72	phylum
Prochlorococcus_marinus_167539	Cyanobacteria	2	2	0	1	38	phylum
Propionibacterium_freudenreichii_754252	Actinobacteria	2	0	0	0	10;11	species
Rhodothermus_marinus_762570	Bacteroidetes	1	12	0	1	13	species
Rivularia_sp._373994	Cyanobacteria	2	2	0	1	38	phylum
Rubrobacter_xylanophilus_266117	Actinobacteria	2	0	0	0	5;6	species
Singulisphaera_acidiphila_886293	Planctomycetes	1	12	0	1	72	phylum
Sorangium_cellulosum_448385	Proteobacteria	1	1	0	1	77	species
Spiroplasma_chrysopicola_1276227	Tenericutes	0	0	0	0	87	class
Spiroplasma_melliferum_1129368	Tenericutes	0	0	0	0	87	class
Staphylococcus_aureus_282458	Firmicutes	2	0	0	0	64	species
Streptomyces_coelicolor_100226	Actinobacteria	2	0	2	0	7;8	species
Thermobaculum_terrenum_525904	undef	2	0	0	0	94	species
Thermodesulfatator_indicus_667014	Thermodesulfobacteria	1	12	0	1	88	species
Thermodesulfobacterium_geofontis_795359	Thermodesulfobacteria	1	12	0	1	89	species
Thermodesulfobium_narugense_747365	Firmicutes	1	12	0	1	61	species
Thermodesulfovibrio_yellowstonii_289376	Nitrospirae	1	12	0	1	70	species
Thermomicrobium_roseum_309801	Chloroflexi	1	12	0	1	35;36	species
Thermus_scotoductus_743525	Deinococcus-Thermus	1	12	0	1	46	species
Thiorhodospira_sibirica_765914	Proteobacteria	1	12	0	1	75	species
Turneriella_parva_869212	Spirochaetes	1	12	0	1	85	species
Verrucomicrobiae_bacterium_382464	Verrucomicrobia	1	12	0	1	91	species
Waddlia_chondrophila_716544	Chlamydiae	1	12	0	1	17-20;24	species
Xenococcus_sp._102125	Cyanobacteria	2	2	0	1	38	phylum
planctomycete_KSU-1_247490	Planctomycetes	1	12	0	1	72	phylum

Table S4: Number of POTRA domains predicted by InterProScan in the majority of the sequences composing each orthologous group (OG) identified as a member of the Omp85/TpsB family.

OG	# POTRA domains
MCLdcw112505	1
MCLdcw114962	1
MCLdcw118160	1
MCLdcw118847	1
MCLdcw119966	1
MCLdcw124459	1
MCLdcw140106	1
MCLdcw1107136	1-2
MCLdcw112421	1-2
MCLdcw117894	1-2
MCLdcw143107	1-2
MCLdcw148130	1-2
MCLdcw112372	2
MCLdcw114621	2
MCLdcw1120017	2-3
MCLdcw114035	2-3
MCLdcw133029	2-3
MCLdcw150224	2-3
MCLdcw139371	2-4
MCLdcw113131	3-4
MCLdcw141083	3-4
MCLdcw182166	3-5
MCLdcw111272	4-5

Table S5: Results of the cross-validation procedure comparing four different models of sequence evolution available in PhyloBayes MPI.

mean score +/- stdev	CAT+GTR+Γ	CAT+Γ	GTR+Γ	LG+Γ
CAT+GTR+Γ	/	1279.9 +/- 116.634	8994.2 +/- 347.355	9317.5 +/- 358.479
CAT+Γ	-1279.9 +/- 116.634	/	7714.3 +/- 391.3	8037.6 +/- 407.53
GTR+Γ	-8994.2 +/- 347.355	-7714.3 +/- 391.3	/	323.3 +/- 58.9441
LG+Γ	-9317.5 +/- 358.479	-8037.6 +/- 407.53	-323.3 +/- 58.9441	/

Table S6: Possible roots for the bacterial domain reported in the phylogenomic literature since 2006. The "root" column corresponds to the group shared with our dataset that is the closest to the root in each source. G = Gamma; ML = maximum likelihood; JTT = Jones-Taylor-Thornton; BI = Bayesian inference; WAG = Whelan and Goldman; SPR = subtree prune-and-regraft; CAT = categories; GTR = Generalized time-reversible; LG = Le & Gascuel; R = free rate; F = Frequencies.

source	root	protein type	# proteins	# species	# AAs	methods and models
Battistuzzi & Hedges 2009	Deinococcus-Thermus	rRNA	not specified	189	3786	Felsenstein 84+G
Battistuzzi & Hedges 2009	Terrabacteria	core protein	25	218	6884	ML JTT+G // BI JTT+G
Forterre 2015 (based on Woese 1990)	PVC	16S rRNA	1	6	Not specified	Jukes-Cantor 69
Yutin et al. 2012	Proteobacteria	ribosomal proteins	50	995	6127	ML WAG+G
Whidden et al. 2014	Deferribacteres/Nitrospira	not specified	40,631	244	N/A	SPR
Raymann et al. 2015	Terrabacteria	core proteins	46	134	10,986	BI CAT+GTR+

						G
Ciccarelli et al. 2006	Firmicutes	universal proteins	31	191	8090	ML JTT+G
Hug et al. 2019	Cyanobacteria	core proteins	120	21,943	34,744	ML WAG+G / LG(+G)
Coleman et al. 2021	Terrabacteria	marker genes	62	265	N/A	LG+C60+R8+F

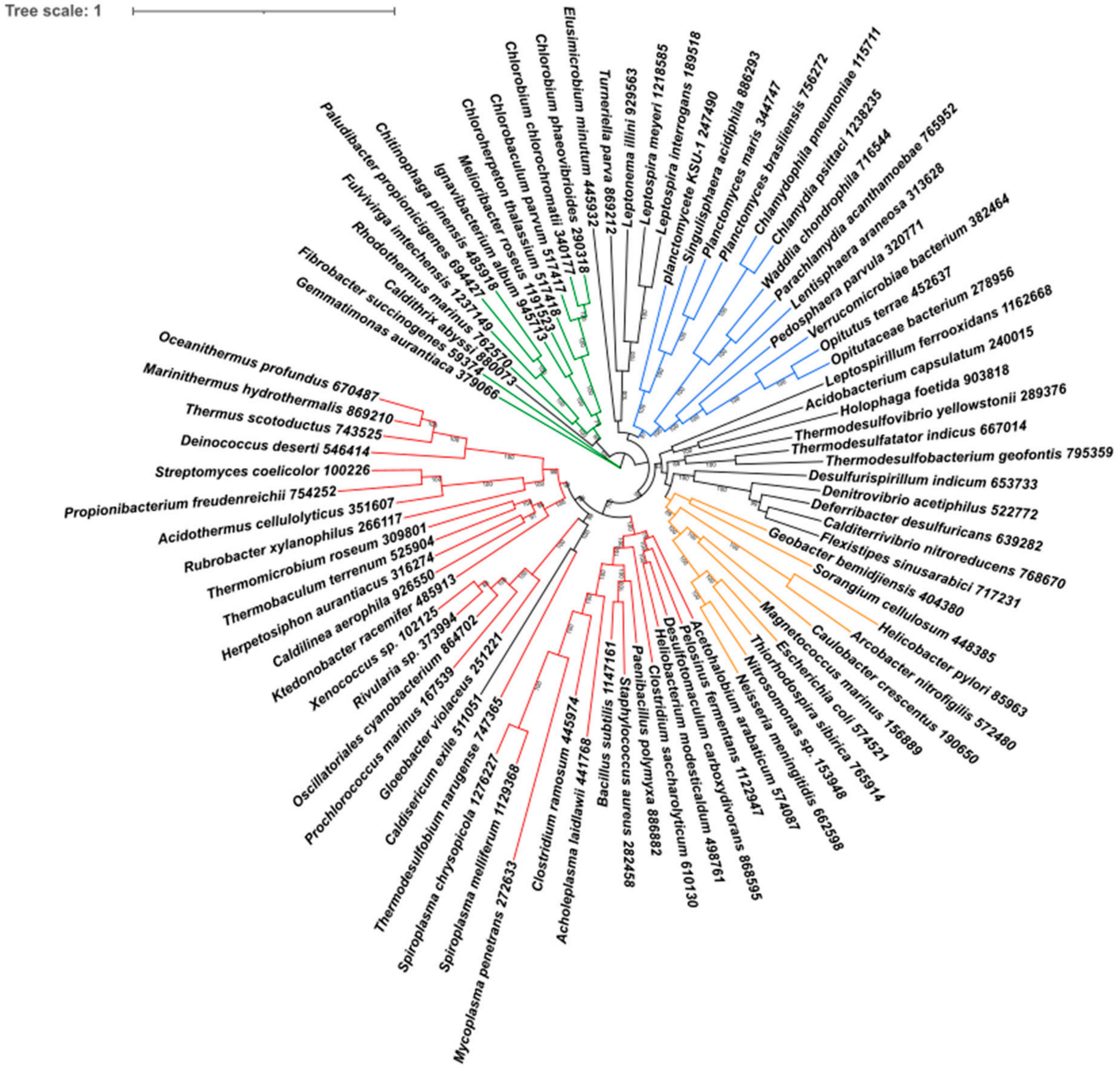

Figure S1: Unrooted phylogenomic tree of the bacterial domain based on a supermatrix concatenating 117 single-copy orthologous genes chosen for their broad conservation across Bacteria. The supermatrix contained 85 species and 19,959 unambiguously aligned amino-acid positions (< 5% missing character states). The tree was inferred from amino-acid sequences using RAxML v8.1.17 with the LG+F+Γ model of sequence evolution. Color key is: red = Terrabacteria, orange = Proteobacteria, green = FBC superphylum, blue = PVC superphylum, black = other.

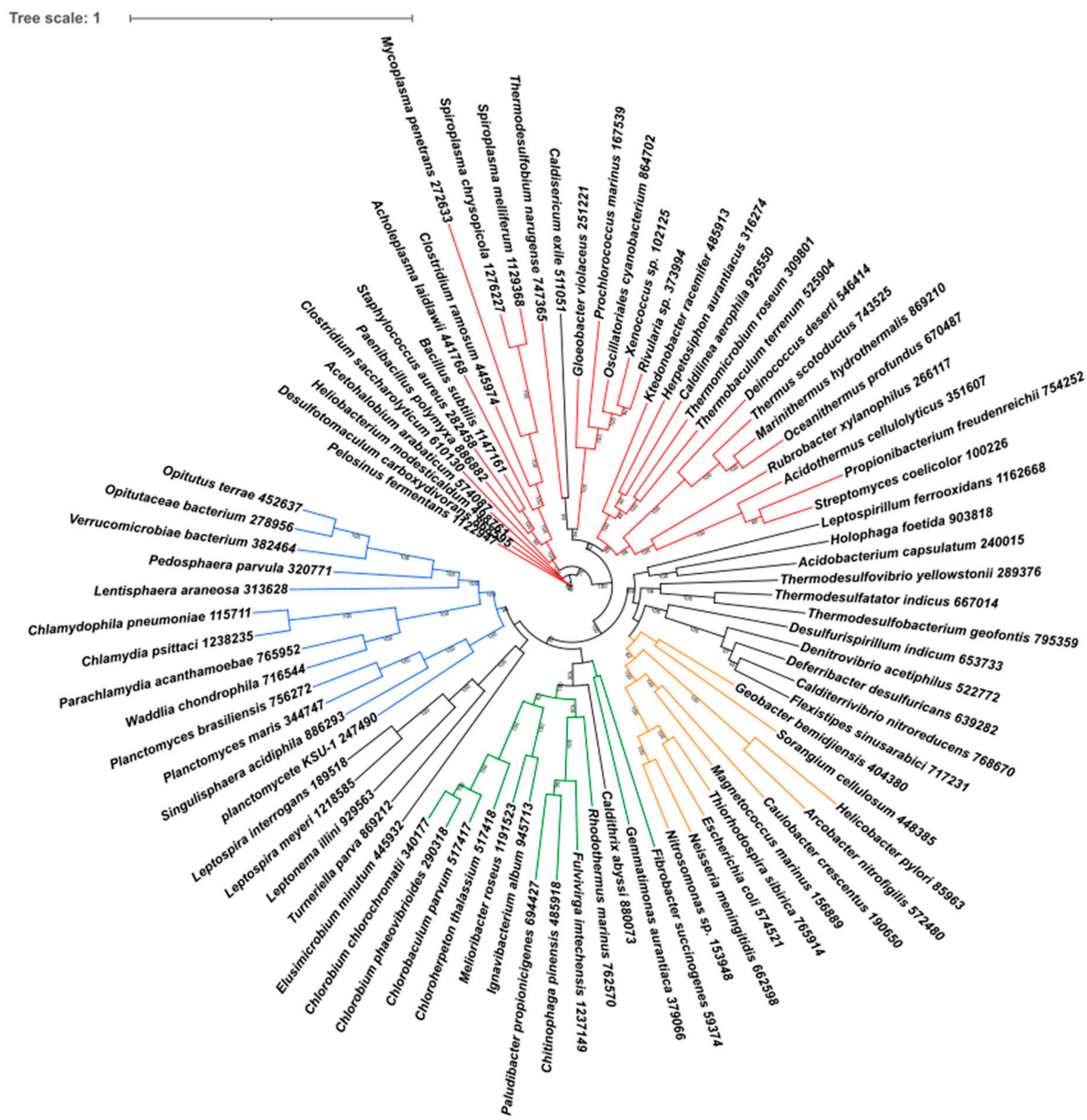

Figure S2: Unrooted phylogenomic tree of the bacterial domain based on a supermatrix concatenating 117 single-copy orthologous genes chosen for their broad conservation across Bacteria. The supermatrix contained 85 species and 19,959 unambiguously aligned amino-acid positions (< 5% missing character states). "Cell-by-cell outliers" (62 sequences) identified by Phylo-MCOA v1.4 were removed from the supermatrix. The tree was inferred from amino-acid sequences using RAxML v8.1.17 with the LG+F+Γ model of sequence evolution.Color key is: red = Terrabacteria, orange = Proteobacteria, green = FBC superphylum, blue = PVC superphylum, black = other.

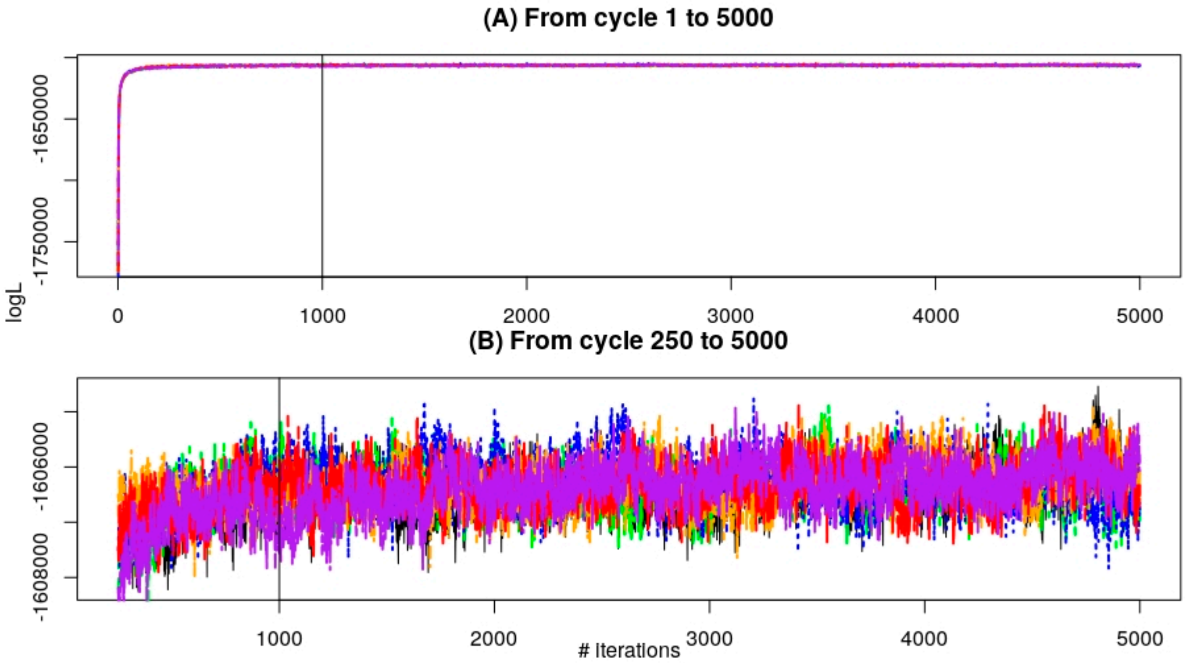

Figure S3: Evolution of the log likelihood of six PhyloBayes MCMC chains running under the CAT+GTR+Γ model of sequence evolution. The vertical line at cycle 1000 marks the end of the burnin. The supermatrix is the one of Figure S1.

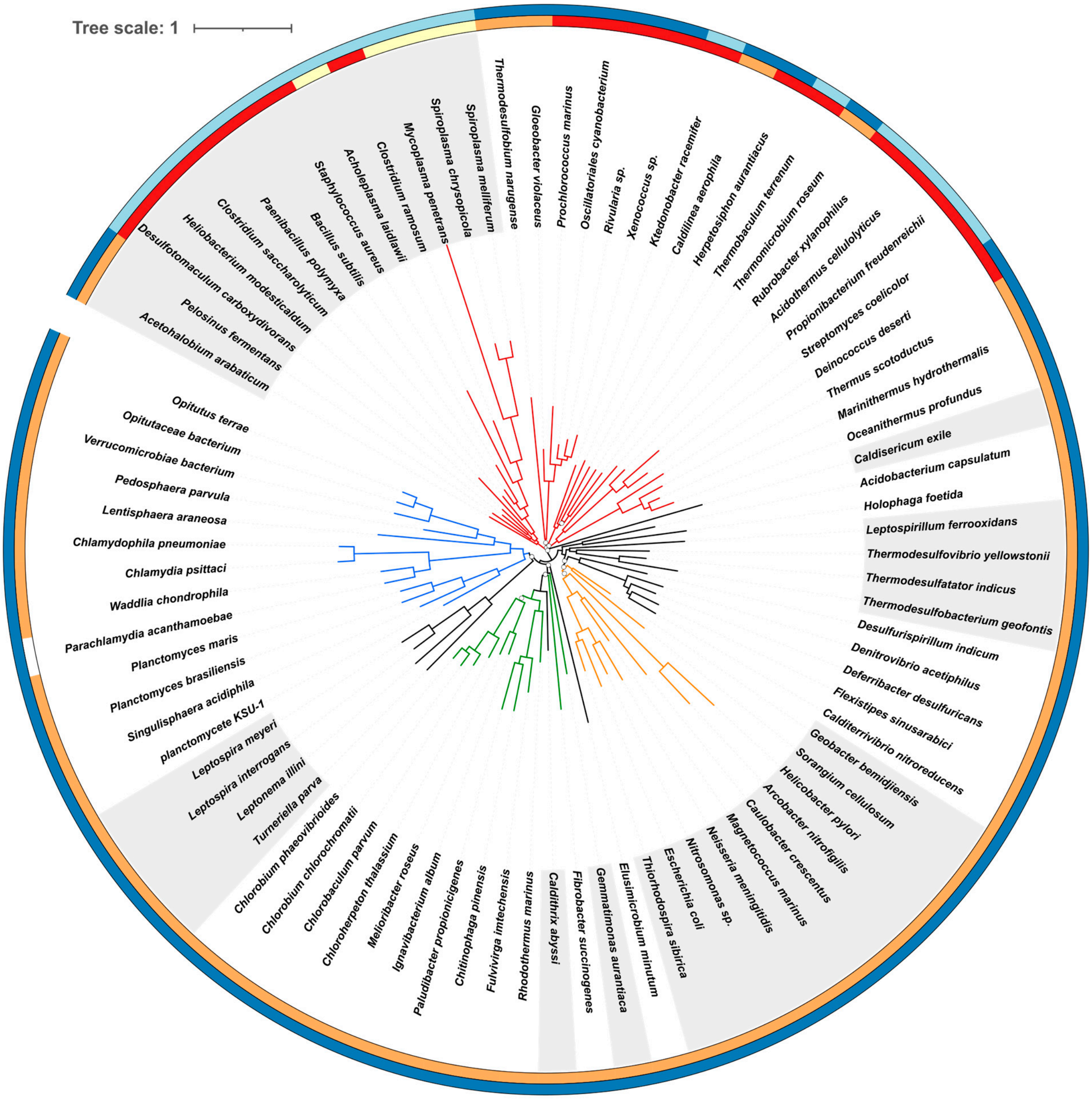

Figure S4: Phylogenomic tree of the bacterial domain based on a supermatrix concatenating 117 single-copy orthologous genes chosen for their broad conservation across Bacteria. This tree is the consensus of the six MCMC chains (A to F) shown in Figure S5. The tree was rooted on the Terrabacteria. Otherwise, it is equivalent to Figure S1. Color key is: red = Terrabacteria, orange = Proteobacteria, green = FBC superphylum, blue = PVC superphylum, black = other. Outer circles represent the status of the peptidoglycan (PG) and of the outer membrane in the organisms, according to our literature survey: red = thick PG, orange = thin PG, yellow = no PG, dark blue = diderm, light blue = monoderm, white = no information. Alternating white and gray backgrounds highlight the alternance between differentially colored groups or phyla.

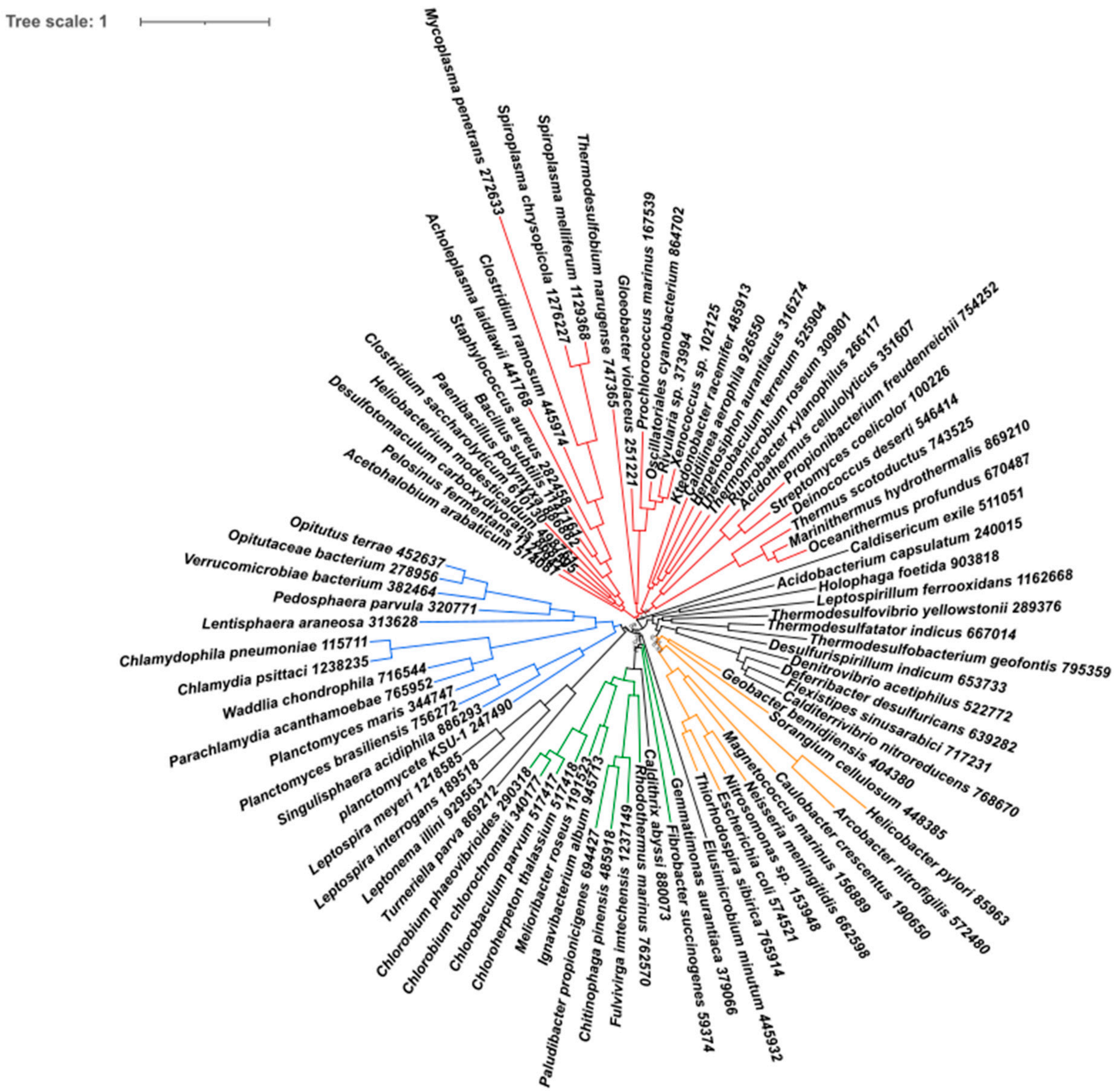
Tree scale: 1
Mycoplasma penetrans 272633
Spiroplasma chrysopicola 1276227
Spiroplasma melliferum 1129368
Thermodesulfobium narugense 747365
Acholeplasma laidlawii 441768
Clostridium ramosum 445974
Staphylococcus aureus
Bacillus subtilis
Paenibacillus polymyxa
Clostridium saccharolyticum
Heliobacterium modesticaldum
Desulfotomaculum carboxydivorans
Pelosinus fermentans
Acetohalobium arabaticum
Gloeobacter violaceus 251221
Prochlorococcus marinus 167539
Oscillatoriales cyanobacterium 864702
Rivularia sp. 373994
Xenococcus sp. 102125
Ktedonobacter racemifer 485913
Caldilinea aerophila 926550
Herpetosiphon aurantiacus 316274
Thermobaculum terrenum 525904
Thermomicrobium roseum 309801
Rubrobacter xylanophilus 266117
Acidothermus cellulolyticus 351607
Propionibacterium freudenreichii 754252
Streptomyces coelicolor 100226
Deinococcus deserti 546414
Thermus scotoductus 743525
Marinithermus hydrothermalis 869210
Oceanithermus profundus 670487
Caldisericum exile 511051
Acidobacterium capsulatum 240015
Holophaga foetida 903818
Leptospirillum ferrooxidans 1162668
Thermodesulfovibrio yellowstonii 289376
Thermodesulfatator indicus 667014
Thermodesulfobacterium geofontis 795359
Desulfurispirillum indicum 653733
Denitrovibrio acetiphilus 522772
Deferribacter desulfuricans 639282
Flexistipes sinusarabici 717231
Calditerrivibrio nitroreducens 768670
Geobacter bemidjiensis 404380
Sorangium cellulosum 448385
Helicobacter pylori 85963
Arcobacter nitrofigilis 572480
Caulobacter crescentus 190650
Magnetococcus marinus 156889
Neisseria meningitidis 662598
Nitrosomonas sp. 153948
Escherichia coli 574521
Thiorhodospira sibirica 765914
Elusimicrobium minutum 445932
Gemmatimonas aurantiaca 379066
Fibrobacter succinogenes 59374
Caldithrix abyssi 880073
Rhodothermus marinus 762570
Fulvivirga imtechensis 1237149
Chitinophaga pinensis 485918
Paludibacter propionicigenes 694427
Ignavibacterium album 945713
Melioribacter roseus 1191523
Chloroherpeton thalassium 517418
Chlorobaculum parvum 517417
Chlorobium chlorochromatii 340177
Chlorobium phaeovibrioides 290318
Turneriella parva 869212
Leptonema illini 929563
Leptospira interrogans 189518
Leptospira meyeri 1218585
planctomycete KSU-1 247490
Singulisphaera acidiphila 886293
Planctomyces brasiliensis 756272
Planctomyces maris 344747
Parachlamydia acanthamoebae 765952
Waddlia chondrophila 716544
Chlamydia psittaci 1238235
Chlamydophila pneumoniae 115711
Lentisphaera araneosa 313628
Pedosphaera parvula 320771
Verrucomicrobiae bacterium 382464
Opitutaceae bacterium 278956
Opitutus terrae 452637

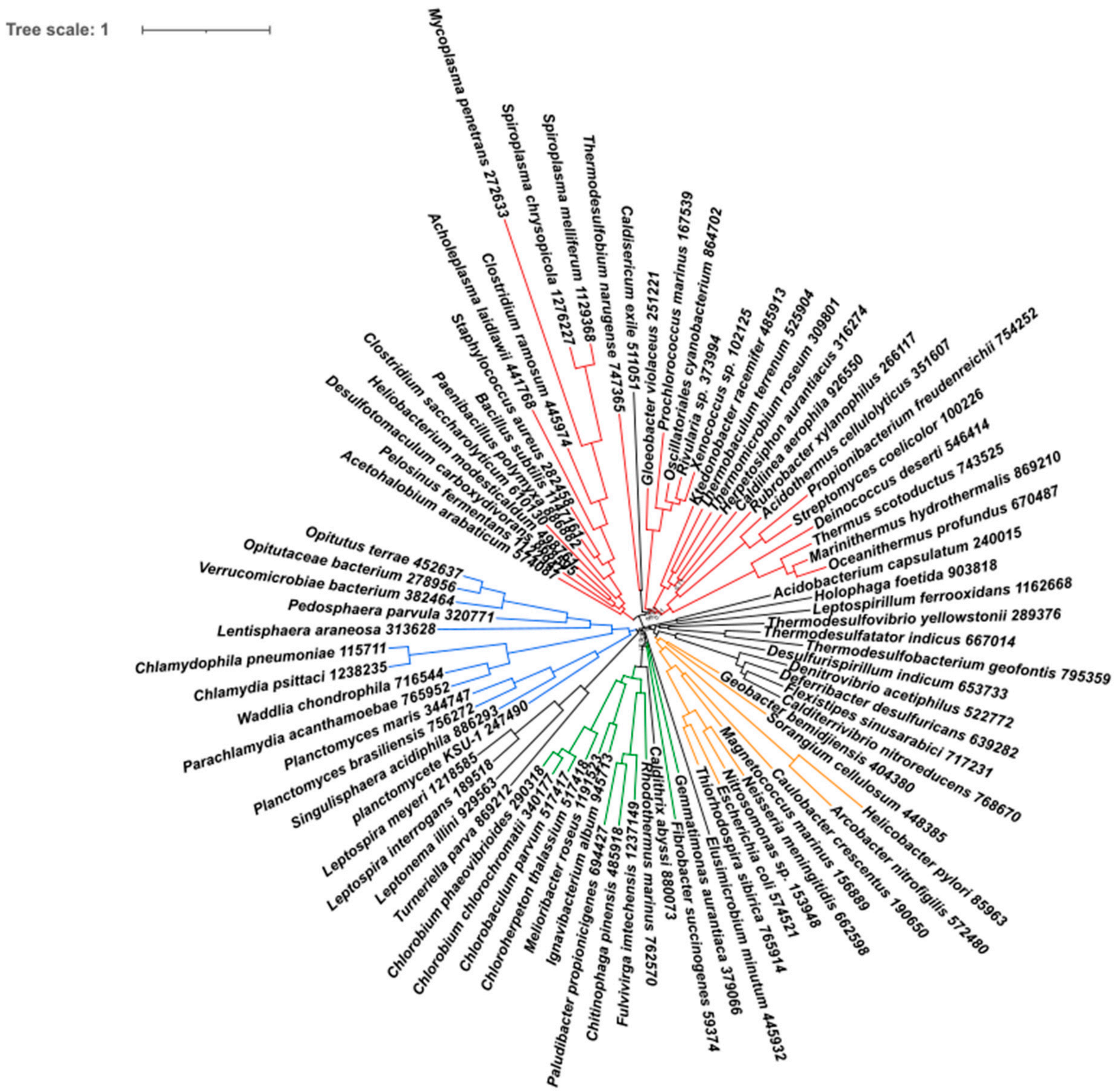

Tree scale: 1
Mycoplasma penetrans 272633
Spiroplasma chrysopicola 1276227
Spiroplasma melliferum 1129368
Thermodesulfobium narugense 747365
Caldisericum exile 511051
Acholeplasma laidlawii 441768
Clostridium ramosum 445974
Staphylococcus aureus 282458
Gloeobacter violaceus 251221
Prochlorococcus marinus 167539
Oscillatoriales cyanobacterium 864702
Rivularia sp. 373994
Xenococcus sp. 102125
Ktedonobacter racemifer 485913
Thermobaculum terrenum 525904
Thermomicrobium roseum 309801
Herpetosiphon aurantiacus 316274
Caldilinea aerophila 926550
Rubrobacter xylanophilus 266117
Acidothermus cellulolyticus 351607
Propionibacterium freudenreichii 754252
Streptomyces coelicolor 100226
Deinococcus deserti 546414
Thermus scotoductus 743525
Marinithermus hydrothermalis 869210
Oceanithermus profundus 670487
Acidobacterium capsulatum 240015
Holophaga foetida 903818
Leptospirillum ferrooxidans 1162668
Thermodesulfovibrio yellowstonii 289376
Thermodesulfatator indicus 667014
Thermodesulfobacterium geofontis 795359
Desulfurispirillum indicum 653733
Denitrovibrio acetiphilus 522772
Deferribacter desulfuricans 639282
Flexistipes sinusarabici 717231
Calditerrivibrio nitroreducens 768670
Geobacter bemidjiensis 404380
Sorangium cellulosum 448385
Helicobacter pylori 85963
Arcobacter nitrofigilis 572480
Caulobacter crescentus 190650
Magnetococcus marinus 156889
Neisseria meningitidis 662598
Nitrosomonas sp. 153948
Escherichia coli 574521
Thiorhodospira sibirica 765914
Elusimicrobium minutum 445932
Gemmatimonas aurantiaca 379066
Fibrobacter succinogenes 59374
Caldithrix abyssi 880073
Rhodothermus marinus 762570
Fulvivirga imtechensis 1237149
Chitinophaga pinensis 485918
Paludibacter propionicigenes 694427
Ignavibacterium album 945713
Melioribacter roseus 1191523
Chloroherpeton thalassium 517418
Chlorobaculum parvum 517417
Chlorobium chlorochromatii 340177
Chlorobium phaeovibrioides 290318
Turneriella parva 869212
Leptonema illini 929563
Leptospira interrogans 189518
Leptospira meyeri 1218585
planctomycete KSU-1 247490
Singulisphaera acidiphila 886293
Planctomyces brasiliensis 756272
Planctomyces maris 344747
Parachlamydia acanthamoebae 765952
Waddlia chondrophila 716544
Chlamydia psittaci 1238235
Chlamydophila pneumoniae 115711
Lentisphaera araneosa 313628
Pedosphaera parvula 320771
Verrucomicrobiae bacterium 382464
Opitutaceae bacterium 278956
Opitutus terrae 452637
Acetohalobium arabaticum 574087
Pelosinus fermentans
Desulfotomaculum carboxydivorans
Heliobacterium modesticaldum
Clostridium saccharolyticum 610130
Paenibacillus polymyxa
Bacillus subtilis

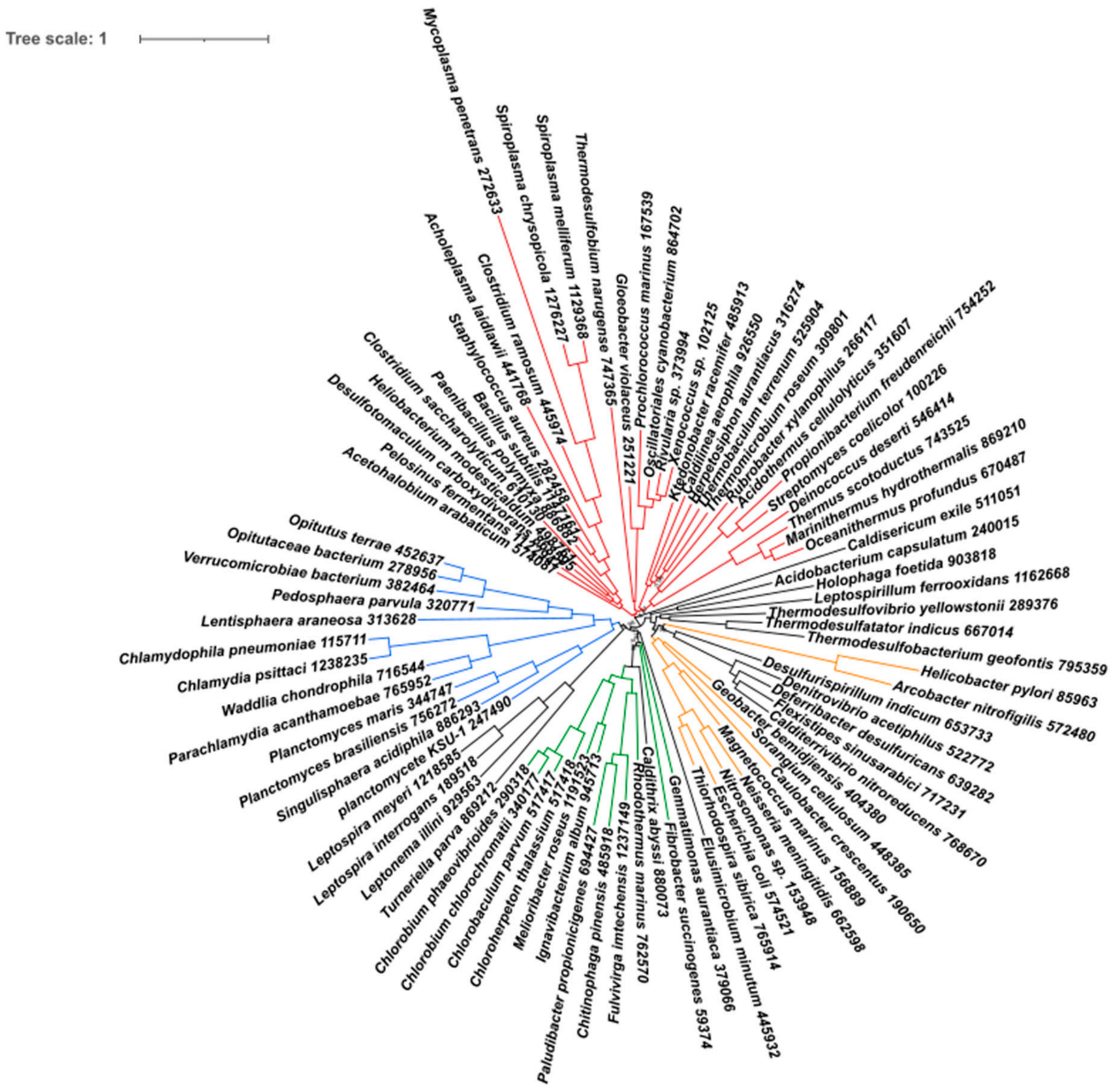

Tree scale: 1
Mycoplasma penetrans 272633
Spiroplasma chrysopicola 1276227
Spiroplasma melliferum 1129368
Thermodesulfobium narugense 747365
Acholeplasma laidlawii 441768
Clostridium ramosum 445974
Staphylococcus aureus 282458
Bacillus subtilis
Paenibacillus polymyxa
Clostridium saccharolyticum
Heliobacterium modesticaldum
Desulfotomaculum carboxydivorans
Pelosinus fermentans
Acetohalobium arabaticum 574087
Gloeobacter violaceus 251221
Prochlorococcus marinus 167539
Oscillatoriales cyanobacterium 864702
Rivularia sp. 373994
Xenococcus sp. 102125
Ktedonobacter racemifer 485913
Caldilinea aerophila 926550
Herpetosiphon aurantiacus 316274
Thermobaculum terrenum 525904
Thermomicrobium roseum 309801
Rubrobacter xylanophilus 266117
Acidothermus cellulolyticus 351607
Propionibacterium freudenreichii 754252
Streptomyces coelicolor 100226
Deinococcus deserti 546414
Thermus scotoductus 743525
Marinithermus hydrothermalis 869210
Oceanithermus profundus 670487
Caldisericum exile 511051
Acidobacterium capsulatum 240015
Holophaga foetida 903818
Leptospirillum ferrooxidans 1162668
Thermodesulfovibrio yellowstonii 289376
Thermodesulfatator indicus 667014
Thermodesulfobacterium geofontis 795359
Helicobacter pylori 85963
Arcobacter nitrofigilis 572480
Desulfurispirillum indicum 653733
Denitrovibrio acetiphilus 522772
Deferribacter desulfuricans 639282
Flexistipes sinusarabici 717231
Calditerrivibrio nitroreducens 768670
Geobacter bemidjiensis 404380
Sorangium cellulosum 448385
Caulobacter crescentus 190650
Magnetococcus marinus 156889
Neisseria meningitidis 662598
Nitrosomonas sp. 153948
Escherichia coli 574521
Thiorhodospira sibirica 765914
Elusimicrobium minutum 445932
Gemmatimonas aurantiaca 379066
Fibrobacter succinogenes 59374
Caldithrix abyssi 880073
Rhodothermus marinus 762570
Fulvivirga imtechensis 1237149
Chitinophaga pinensis 485918
Paludibacter propionicigenes 694427
Ignavibacterium album 945713
Melioribacter roseus 1191523
Chloroherpeton thalassium 517418
Chlorobaculum parvum 517417
Chlorobium chlorochromatii 340177
Chlorobium phaeovibrioides 290318
Turneriella parva 869212
Leptonema illini 929563
Leptospira interrogans 189518
Leptospira meyeri 1218585
planctomycete KSU-1 247490
Singulisphaera acidiphila 886293
Planctomyces brasiliensis 756272
Planctomyces maris 344747
Parachlamydia acanthamoebae 765952
Waddlia chondrophila 716544
Chlamydia psittaci 1238235
Chlamydophila pneumoniae 115711
Lentisphaera araneosa 313628
Pedosphaera parvula 320771
Verrucomicrobiae bacterium 382464
Opitutaceae bacterium 278956
Opitutus terrae 452637

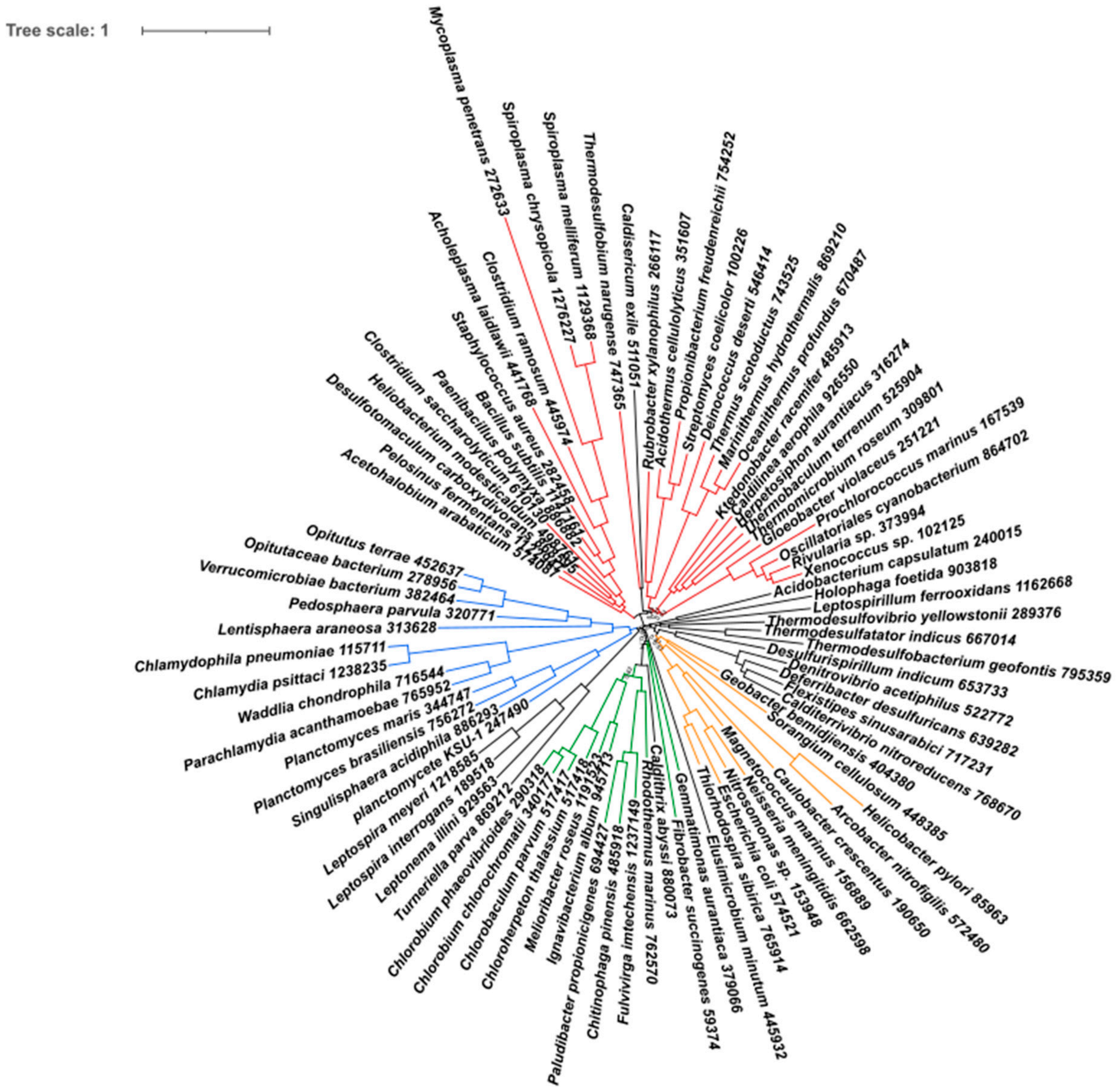
Tree scale: 1
Mycoplasma penetrans 272633
Spiroplasma chrysopicola 1276227
Spiroplasma melliferum 1129368
Thermodesulfobium narugense 747365
Caldisericum exile 511051
Rubrobacter xylanophilus 266117
Acidothermus cellulolyticus 351607
Propionibacterium freudenreichii 754252
Streptomyces coelicolor 100226
Deinococcus deserti 546414
Thermus scotoductus 743525
Marinithermus hydrothermalis 869210
Oceanithermus profundus 670487
Ktedonobacter racemifer 485913
Caldilinea aerophila 926550
Herpetosiphon aurantiacus 316274
Thermobaculum terrenum 525904
Thermomicrobium roseum 309801
Gloeobacter violaceus 251221
Prochlorococcus marinus 167539
Oscillatoriales cyanobacterium 864702
Rivularia sp. 373994
Xenococcus sp. 102125
Acidobacterium capsulatum 240015
Holophaga foetida 903818
Leptospirillum ferrooxidans 1162668
Thermodesulfovibrio yellowstonii 289376
Thermodesulfatator indicus 667014
Thermodesulfobacterium geofontis 795359
Desulfurispirillum indicum 653733
Denitrovibrio acetiphilus 522772
Deferribacter desulfuricans 639282
Flexistipes sinusarabici 717231
Calditerrivibrio nitroreducens 768670
Geobacter bemidjiensis 404380
Sorangium cellulosum 448385
Helicobacter pylori 85963
Arcobacter nitrofigilis 572480
Caulobacter crescentus 190650
Magnetococcus marinus 156889
Neisseria meningitidis 662598
Nitrosomonas sp. 153948
Escherichia coli 574521
Thiorhodospira sibirica 765914
Elusimicrobium minutum 445932
Gemmatimonas aurantiaca 379066
Fibrobacter succinogenes 59374
Caldithrix abyssi 880073
Rhodothermus marinus 762570
Fulvivirga imtechensis 1237149
Chitinophaga pinensis 485918
Paludibacter propionicigenes 694427
Ignavibacterium album 945713
Melioribacter roseus 1191523
Chloroherpeton thalassium 517418
Chlorobaculum parvum 517417
Chlorobium chlorochromatii 340177
Chlorobium phaeovibrioides 290318
Turneriella parva 869212
Leptonema illini 929563
Leptospira interrogans 189518
Leptospira meyeri 1218585
planctomycete KSU-1 247490
Singulisphaera acidiphila 886293
Planctomyces brasiliensis 756272
Planctomyces maris 344747
Parachlamydia acanthamoebae 765952
Waddlia chondrophila 716544
Chlamydia psittaci 1238235
Chlamydophila pneumoniae 115711
Lentisphaera araneosa 313628
Pedosphaera parvula 320771
Verrucomicrobiae bacterium 382464
Opitutaceae bacterium 278956
Opitutus terrae 452637
Acetohalobium arabaticum 574087
Pelosinus fermentans
Desulfotomaculum carboxydivorans
Heliobacterium modesticaldum
Clostridium saccharolyticum 610130
Paenibacillus polymyxa
Bacillus subtilis
Staphylococcus aureus 282458
Clostridium ramosum 445974
Acholeplasma laidlawii 441768

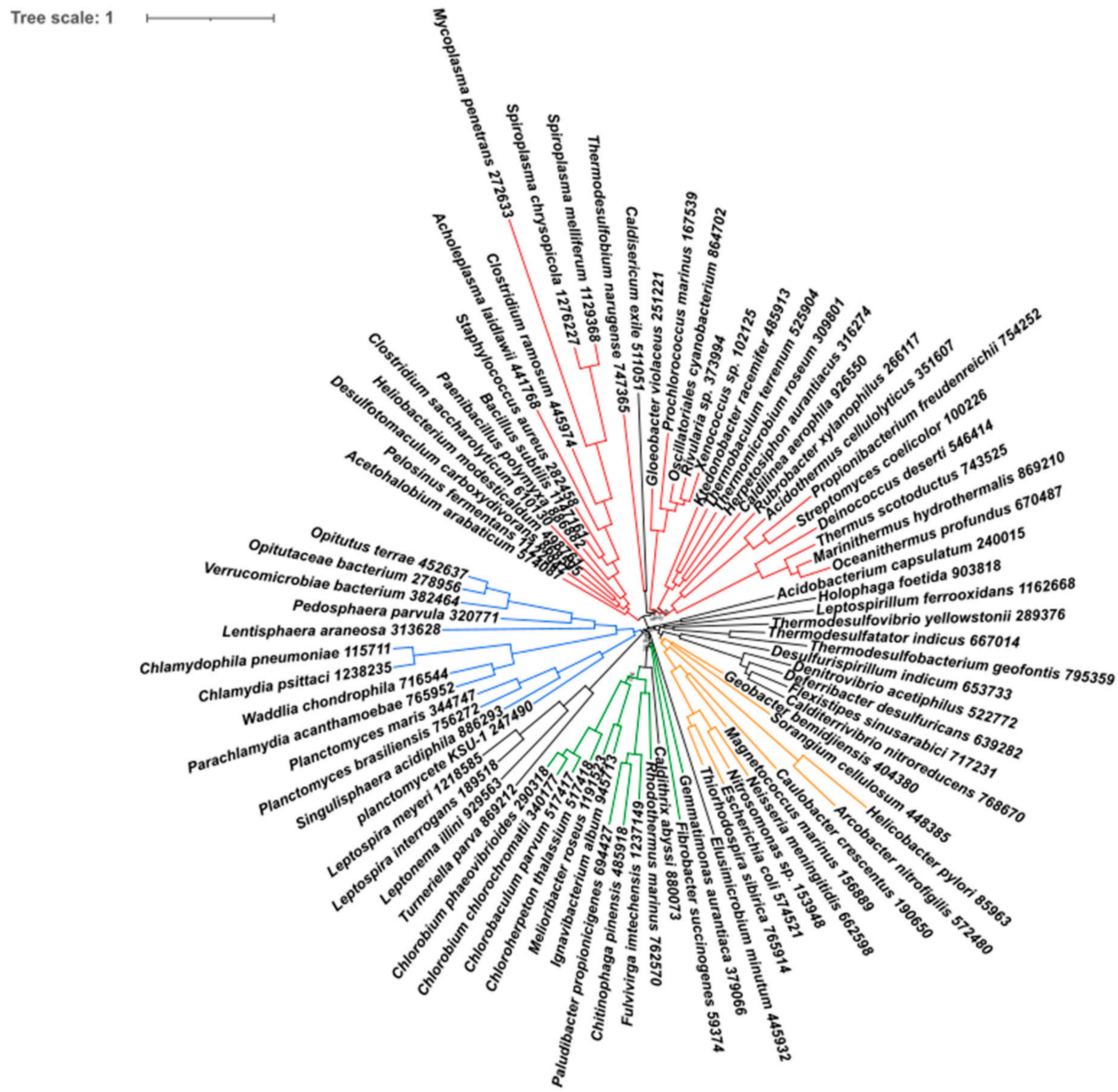

Tree scale: 1
Mycoplasma penetrans 272633
Spiroplasma chrysopicola 1276227
Spiroplasma melliferum 1129368
Thermodesulfobium narugense 747365
Caldisericum exile 511051
Acholeplasma laidlawii 441768
Clostridium ramosum 445974
Staphylococcus aureus 282458
Bacillus subtilis
Paenibacillus polymyxa
Clostridium saccharolyticum 610130
Heliobacterium modesticaldum
Desulfotomaculum carboxydivorans
Pelosinus fermentans
Acetohalobium arabaticum 574087
Gloeobacter violaceus 251221
Prochlorococcus marinus 167539
Oscillatoriales cyanobacterium 864702
Rivularia sp. 373994
Xenococcus sp. 102125
Ktedonobacter racemifer 485913
Thermobaculum terrenum 525904
Thermomicrobium roseum 309801
Herpetosiphon aurantiacus 316274
Caldilinea aerophila 926550
Rubrobacter xylanophilus 266117
Acidothermus cellulolyticus 351607
Propionibacterium freudenreichii 754252
Streptomyces coelicolor 100226
Deinococcus deserti 546414
Thermus scotoductus 743525
Marinithermus hydrothermalis 869210
Oceanithermus profundus 670487
Acidobacterium capsulatum 240015
Holophaga foetida 903818
Leptospirillum ferrooxidans 1162668
Thermodesulfovibrio yellowstonii 289376
Thermodesulfatator indicus 667014
Thermodesulfobacterium geofontis 795359
Desulfurispirillum indicum 653733
Denitrovibrio acetiphilus 522772
Deferribacter desulfuricans 639282
Flexistipes sinusarabici 717231
Calditerrivibrio nitroreducens 768670
Geobacter bemidjiensis 404380
Sorangium cellulosum 448385
Helicobacter pylori 85963
Arcobacter nitrofigilis 572480
Caulobacter crescentus 190650
Magnetococcus marinus 156889
Neisseria meningitidis 662598
Nitrosomonas sp. 153948
Escherichia coli 574521
Thiorhodospira sibirica 765914
Elusimicrobium minutum 445932
Gemmatimonas aurantiaca 379066
Fibrobacter succinogenes 59374
Caldithrix abyssi 880073
Rhodothermus marinus 762570
Fulvivirga imtechensis 1237149
Chitinophaga pinensis 485918
Paludibacter propionicigenes 694427
Ignavibacterium album 945713
Melioribacter roseus 1191523
Chloroherpeton thalassium 517418
Chlorobaculum parvum 517417
Chlorobium chlorochromatii 340177
Chlorobium phaeovibrioides 290318
Turneriella parva 869212
Leptonema illini 929563
Leptospira interrogans 189518
Leptospira meyeri 1218585
planctomycete KSU-1 247490
Singulisphaera acidiphila 886293
Planctomyces brasiliensis 756272
Planctomyces maris 344747
Parachlamydia acanthamoebae 765952
Waddlia chondrophila 716544
Chlamydia psittaci 1238235
Chlamydophila pneumoniae 115711
Lentisphaera araneosa 313628
Pedosphaera parvula 320771
Verrucomicrobiae bacterium 382464
Opitutaceae bacterium 278956
Opitutus terrae 452637

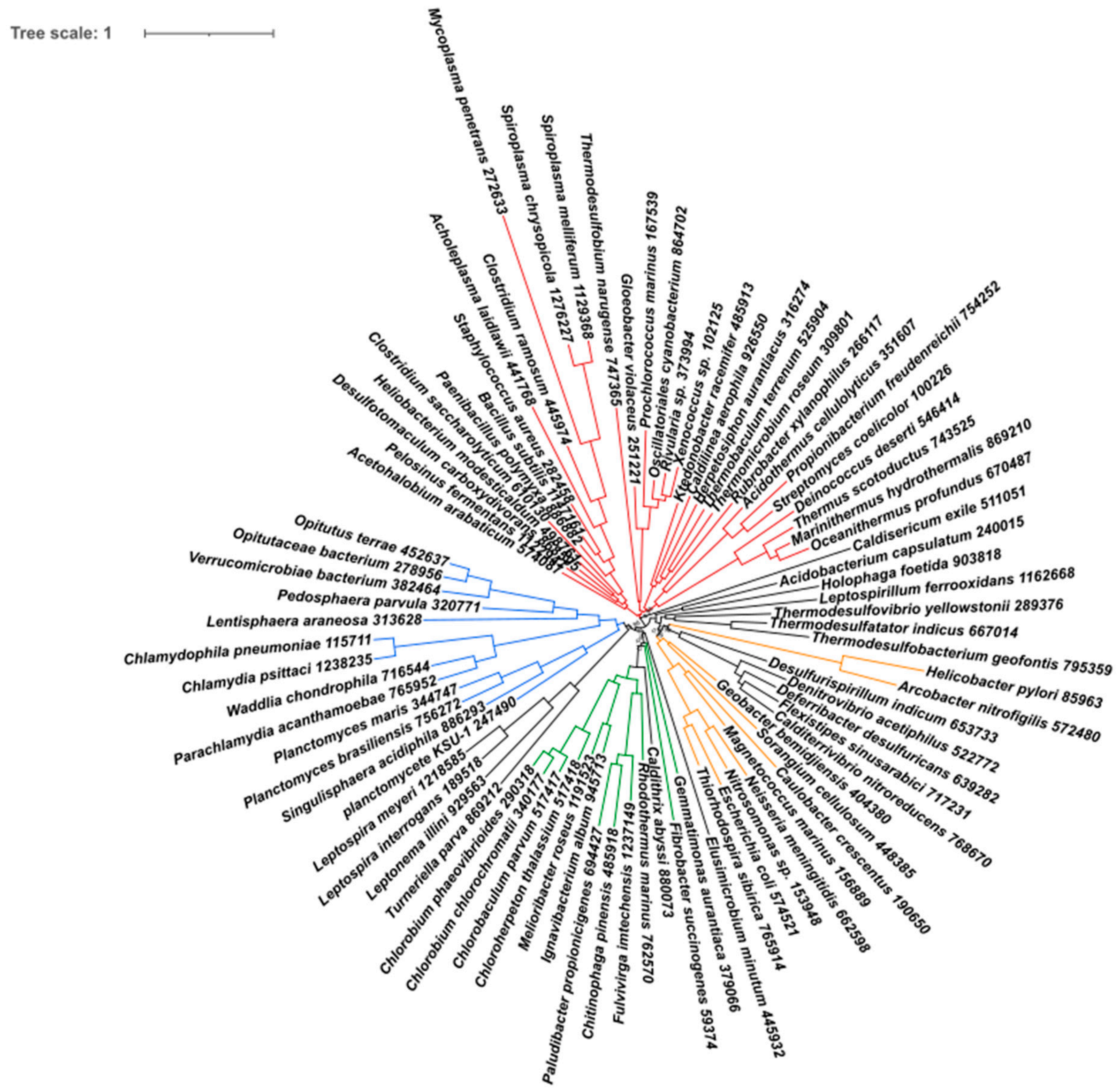

Figure S5: Trees inferred by the six individual MCMC chains running under the CAT+GTR+Γ model of sequence evolution. Trees were rooted on the Terrabacteria. Only posterior probabilities <1.0 are shown. Figure S1 is the consensus of chains A and C. For a consensus built from all six chains, see Figure S4. Color key is: red = Terrabacteria, orange = Proteobacteria, green = FBC superphylum, blue = PVC superphylum, black = other.

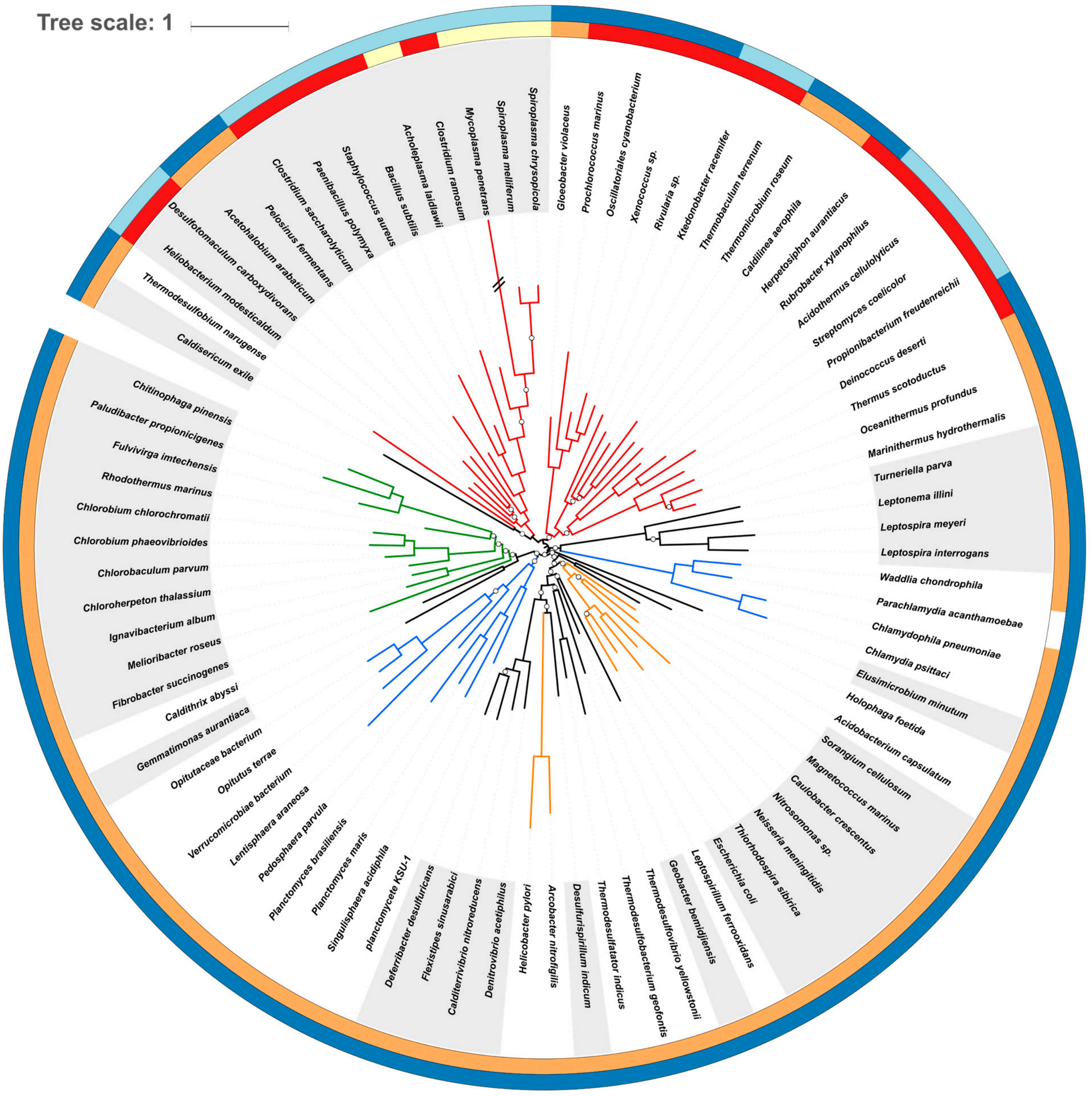

Figure S6: Phylogenomic tree of the bacterial domain based on a supermatrix concatenating 117 single-copy orthologous genes chosen for their broad conservation across Bacteria. The tree was rooted on the Terrabacteria. The supermatrix contained 101 species and 19,959 unambiguously aligned amino-acid positions (4.72% missing character states). The tree was inferred from amino-acid sequences using PhyloBayes MPI and the CAT+Γ model of sequence evolution. Tree annotations are as in Figure S1: the circles at the nodes are posterior probabilities (PP) which are below the maximum statistical support (PP of 1.0). Nodes without a circle correspond to maximum statistical support for phylogenetic inference (PP of 1.0). The branch with "//" means that this branch has been cropped of half its length for clarity. The outer circles represent the status of the peptidoglycan (PG) and the outer membrane (OM) in the organism. Color key is: red = Terrabacteria, orange = Proteobacteria, green = FBC superphylum, blue = PVC superphylum, black = other. Outer circles represent the status of the PG and of the OM in the organisms, according to our literature survey:

red = thick PG, orange = thin PG, yellow = no PG, dark blue = diderm, light blue = monoderm, white = no information. Alternating white and gray backgrounds highlight the alternance between differentially colored groups or phyla.

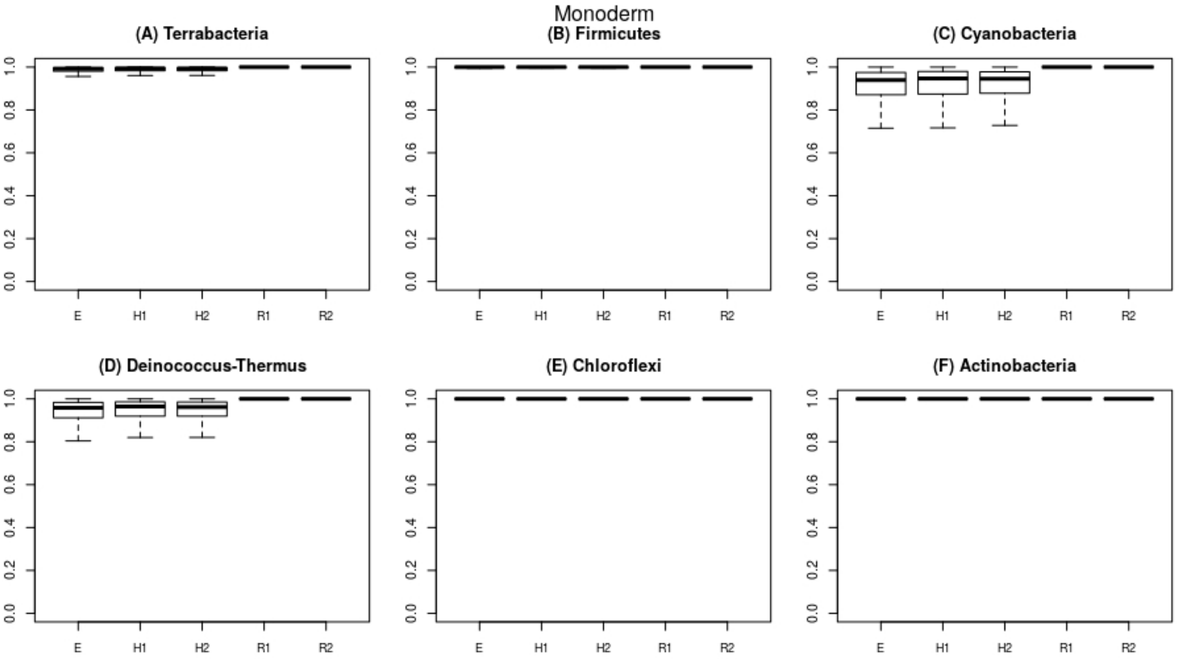

Figure S7: Posterior probabilities for a monoderm LBCA according to five different models, prior exponential of 10 (E), hyperprior exponential 0 to 10 (H1), hyperprior exponential 0 to 100 (H2),reverse jump hyperprior exponential 0 to 10 (R1) and reverse jump hyperprior exponential 0 to 100 (R2), and six possible roots for the bacterial domain (Terrabacteria, Firmicutes, Cyanobacteria, Deinococcus-Thermus, Chloroflexi and Actinobacteria).

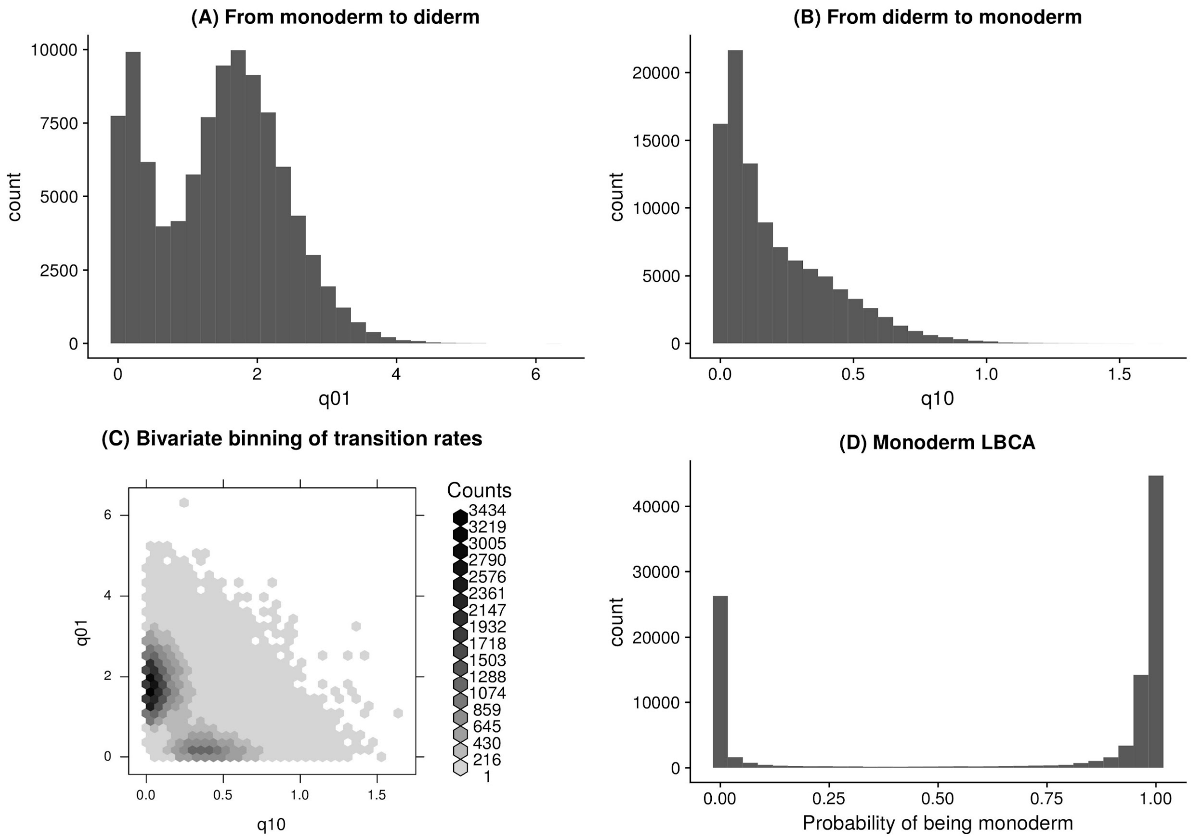

Figure S8: Posterior transition rates and posterior probability of being monoderm for the model where the hyper-prior was purposely biased towards the "diderm-first" hypothesis. "q01" is the transition rate from monoderm to diderm (limited) and "q10" is the transition rate from diderm to monoderm (favored). LBCA = Last Bacterial Common Ancestor.

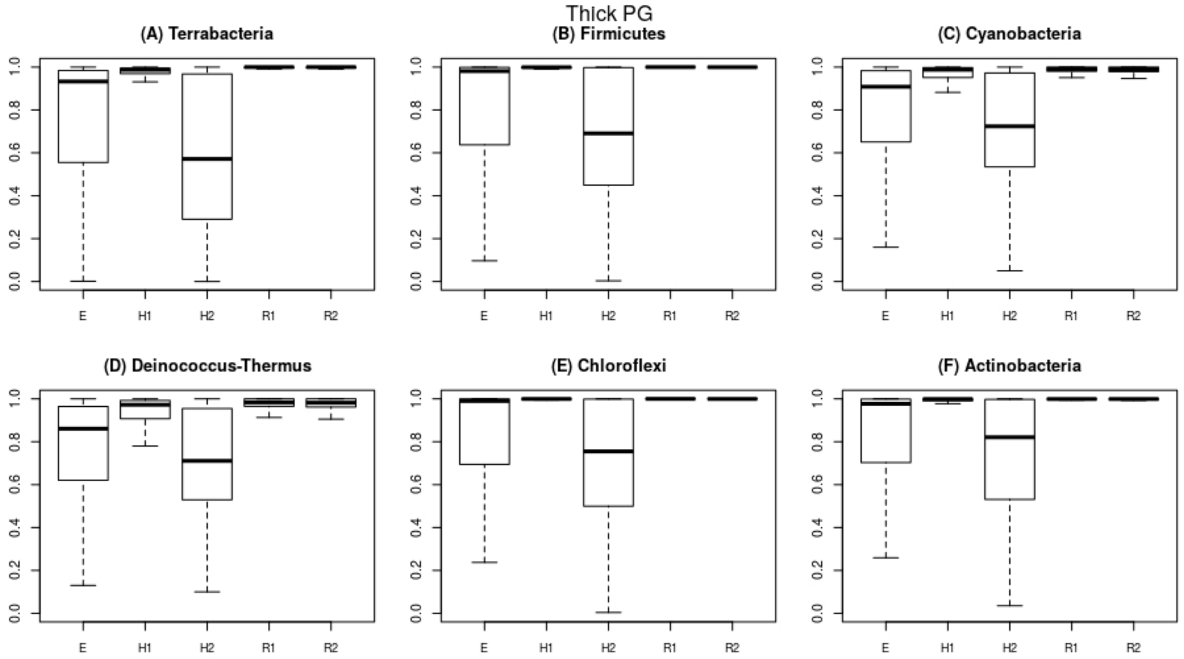

Figure S9: Posterior probabilities for a LBCA featuring a thick peptidoglycan (PG) layer according to the five different models and the six possible bacterial roots.

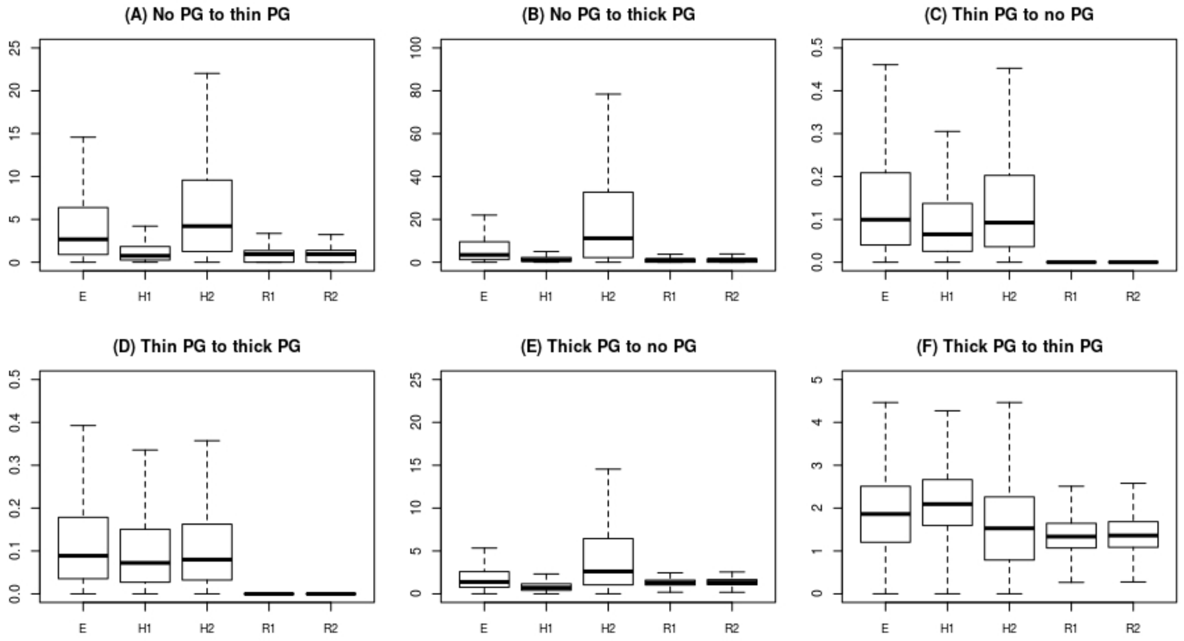

Figure S10: Posterior transition rates for the peptidoglycan (PG) trait. The Terrabacteria root was used for the five models.

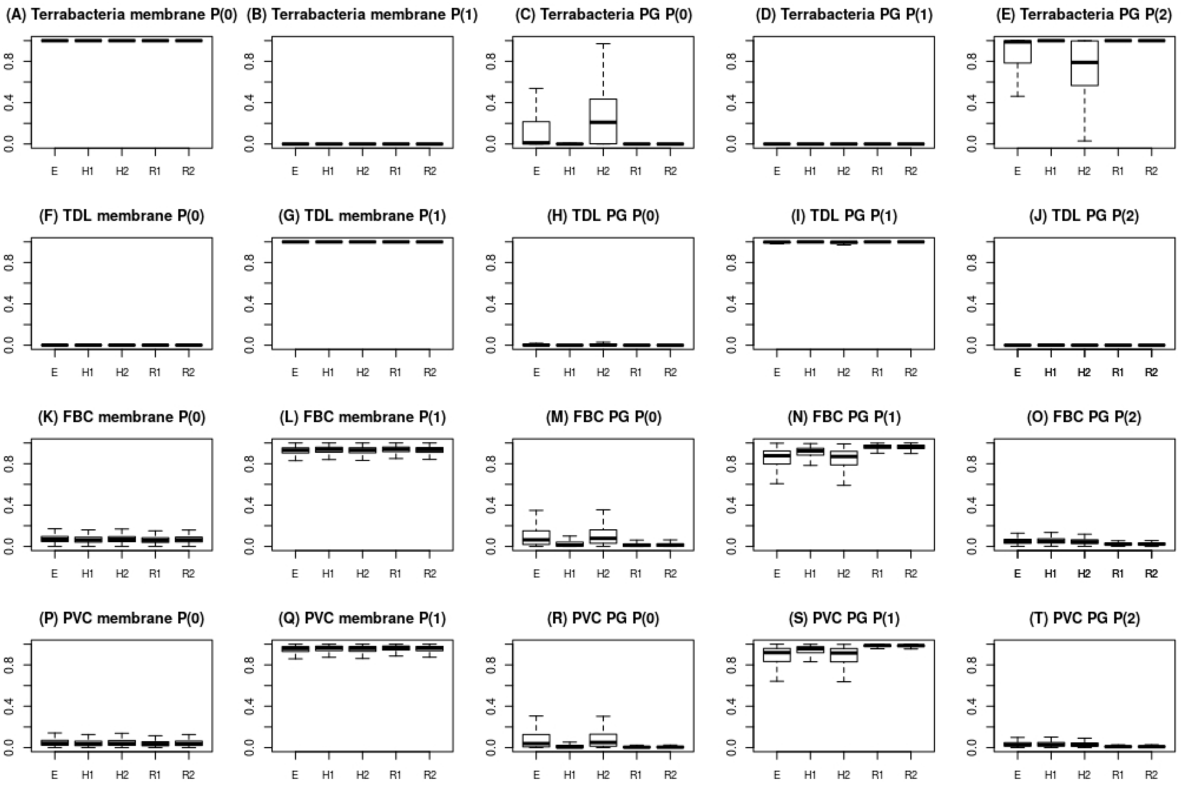

Figure S11: Posterior probabilities for the peptidoglycan (PG) and membrane traits in the LCA of four bacterial groups. Membrane P(0) and P(1) correspond to one and two membranes, respectively, PG P(0), P(1) and P(2) correspond to no PG, thin PG and thick PG, respectively. The Terrabacteria root was used for the five models.

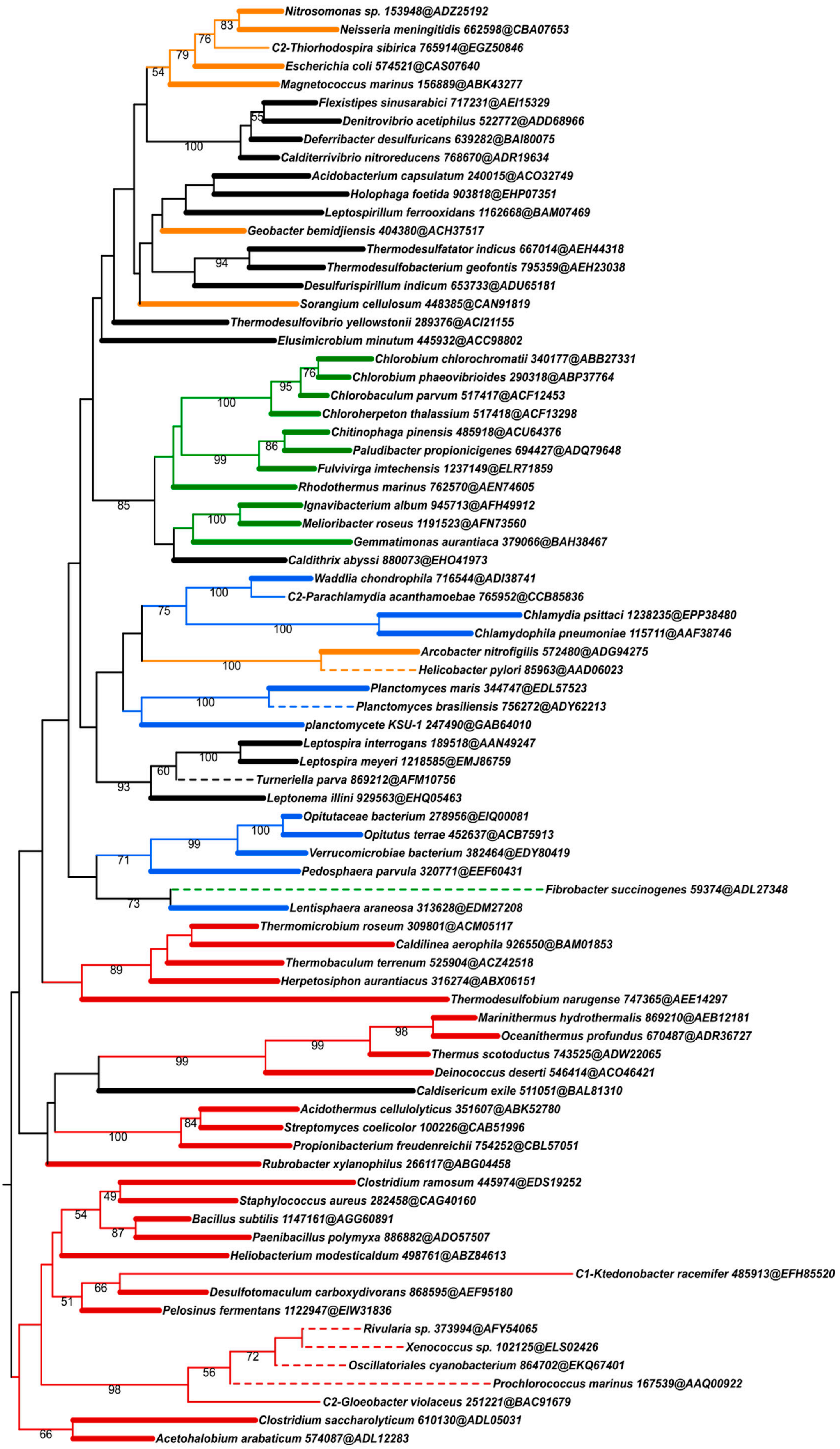
Nitrosomonas sp. 153948@ADZ25192
Neisseria meningitidis 662598@CBA07653
C2-Thiorhodospira sibirica 765914@EGZ50846
Escherichia coli 574521@CAS07640
Magnetococcus marinus 156889@ABK43277
Flexistipes sinusarabici 717231@AEI15329
Denitrovibrio acetiphilus 522772@ADD68966
Deferribacter desulfuricans 639282@BAI80075
Calditerrivibrio nitroreducens 768670@ADR19634
Acidobacterium capsulatum 240015@ACO32749
Holophaga foetida 903818@EHP07351
Leptospirillum ferrooxidans 1162668@BAM07469
Geobacter bemidjiensis 404380@ACH37517
Thermodesulfatator indicus 667014@AEH44318
Thermodesulfobacterium geofontis 795359@AEH23038
Desulfurispirillum indicum 653733@ADU65181
Sorangium cellulosum 448385@CAN91819
Thermodesulfovibrio yellowstonii 289376@ACI21155
Elusimicrobium minutum 445932@ACC98802
Chlorobium chlorochromatii 340177@ABB27331
Chlorobium phaeovibrioides 290318@ABP37764
Chlorobaculum parvum 517417@ACF12453
Chloroherpeton thalassium 517418@ACF13298
Chitinophaga pinensis 485918@ACU64376
Paludibacter propionicigenes 694427@ADQ79648
Fulvivirga imtechensis 1237149@ELR71859
Rhodothermus marinus 762570@AEN74605
Ignavibacterium album 945713@AFH49912
Melioribacter roseus 1191523@AFN73560
Gemmatimonas aurantiaca 379066@BAH38467
Caldithrix abyssi 880073@EHO41973
Waddlia chondrophila 716544@ADI38741
C2-Parachlamydia acanthamoebae 765952@CCB85836
Chlamydia psittaci 1238235@EPP38480
Chlamydophila pneumoniae 115711@AAF38746
Arcobacter nitrofigilis 572480@ADG94275
Helicobacter pylori 85963@AAD06023
Planctomyces maris 344747@EDL57523
Planctomyces brasiliensis 756272@ADY62213
planctomycete KSU-1 247490@GAB64010
Leptospira interrogans 189518@AAN49247
Leptospira meyeri 1218585@EMJ86759
Turneriella parva 869212@AFM10756
Leptonema illini 929563@EHQ05463
Opitutaceae bacterium 278956@EIQ00081
Opitutus terrae 452637@ACB75913
Verrucomicrobiae bacterium 382464@EDY80419
Pedosphaera parvula 320771@EEF60431
Fibrobacter succinogenes 59374@ADL27348
Lentisphaera araneosa 313628@EDM27208
Thermomicrobium roseum 309801@ACM05117
Caldilinea aerophila 926550@BAM01853
Thermobaculum terrenum 525904@ACZ42518
Herpetosiphon aurantiacus 316274@ABX06151
Thermodesulfobium narugense 747365@AEE14297
Marinithermus hydrothermalis 869210@AEB12181
Oceanithermus profundus 670487@ADR36727
Thermus scotoductus 743525@ADW22065
Deinococcus deserti 546414@ACO46421
Caldisericum exile 511051@BAL81310
Acidothermus cellulolyticus 351607@ABK52780
Streptomyces coelicolor 100226@CAB51996
Propionibacterium freudenreichii 754252@CBL57051
Rubrobacter xylanophilus 266117@ABG04458
Clostridium ramosum 445974@EDS19252
Staphylococcus aureus 282458@CAG40160
Bacillus subtilis 1147161@AGG60891
Paenibacillus polymyxa 886882@ADO57507
Heliobacterium modesticaldum 498761@ABZ84613
C1-Ktedonobacter racemifer 485913@EFH85520
Desulfotomaculum carboxydivorans 868595@AEF95180
Pelosinus fermentans 1122947@EIW31836
Rivularia sp. 373994@AFY54065
Xenococcus sp. 102125@ELS02426
Oscillatoriales cyanobacterium 864702@EKQ67401
Prochlorococcus marinus 167539@AAQ00922
C2-Gloeobacter violaceus 251221@BAC91679
Clostridium saccharolyticum 610130@ADL05031
Acetohalobium arabaticum 574087@ADL12283

Figure S12: (previous page) MraY tree inferred using RAxML v8.1.17 under the LG+F+Γ model of sequence evolution (see "DCW_17_SG.pdf", folder Trees, for the remaining *dcw* trees). Tree rooted on the Terrabacteria. Thick branches indicate a gene present in the main cluster (longest cluster), while thin branches indicate a gene present in a sub-cluster (the different sub-clusters are numbered following the nomenclature "Cn" with "n" being the number of the cluster) and dotted branches indicate a gene located outside of any cluster. Color key is: red = Terrabacteria, orange = Proteobacteria, green = FBC superphylum, blue = PVC superphylum, black = other.

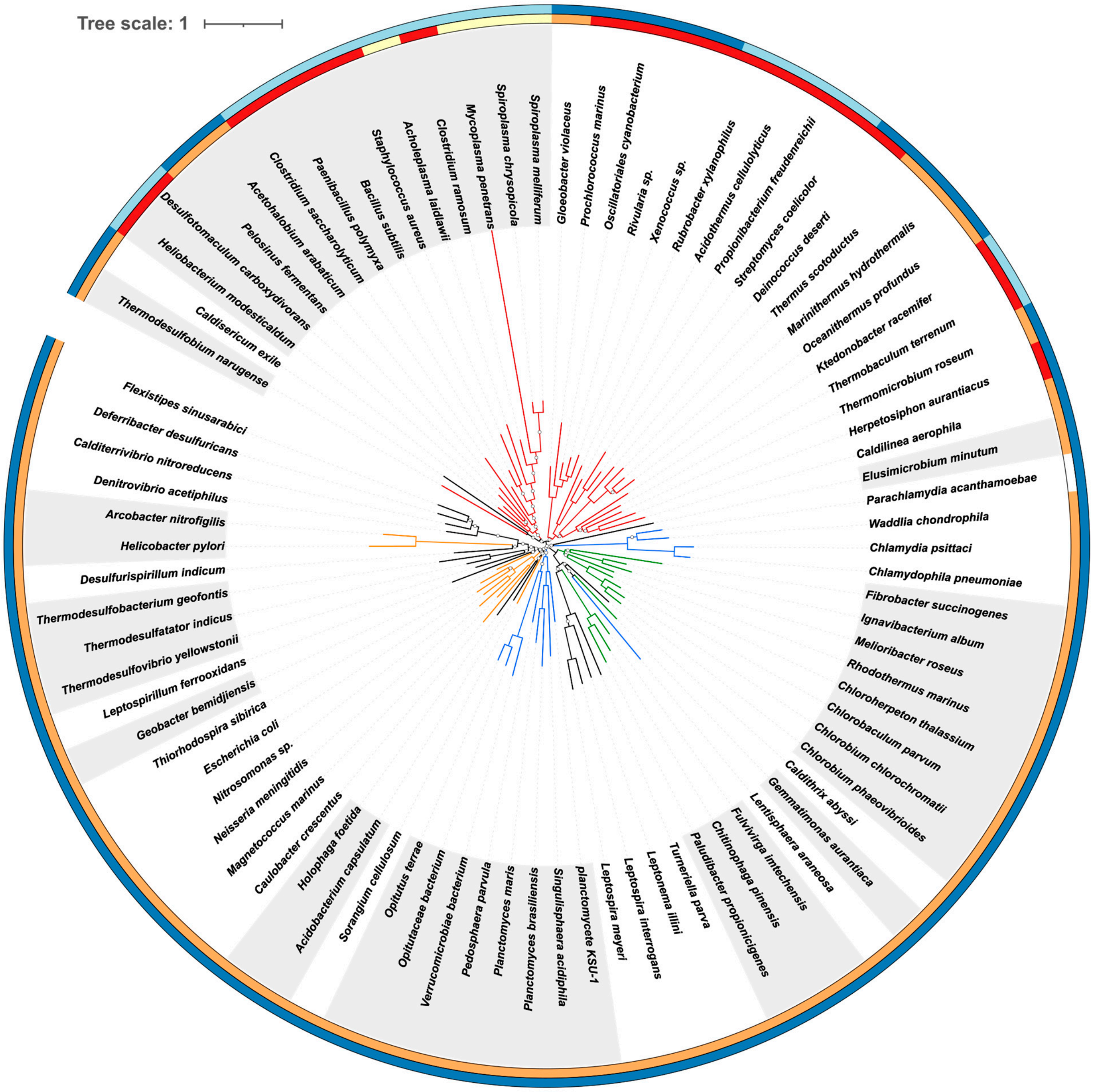

Figure S13: Phylogenomic tree based on a supermatrix of 85 species x 4571 unambiguously aligned amino-acid positions (8.47% missing character states) using 15 of the *dcw* cluster genes. Tree rooted on the Terrabacteria. PhyloBayes MPI v1.4

was used to run two MCMC chains under the CAT+Γ model for 50,000 cycles. Both chains were used to compute the consensus tree (maxdiff = 0.284; meandiff = 0.007). Color key is: red = Terrabacteria, orange = Proteobacteria, green = FBC superphylum, blue = PVC superphylum, black = other. Outer circles represent the status of the peptidoglycan (PG) and of the outer membrane in the organisms, according to our literature survey: red = thick PG, orange = thin PG, yellow = no PG, dark blue = diderm, light blue = monoderm, white = no information. Alternating white and gray backgrounds highlight the alternance between differentially colored groups or phyla.

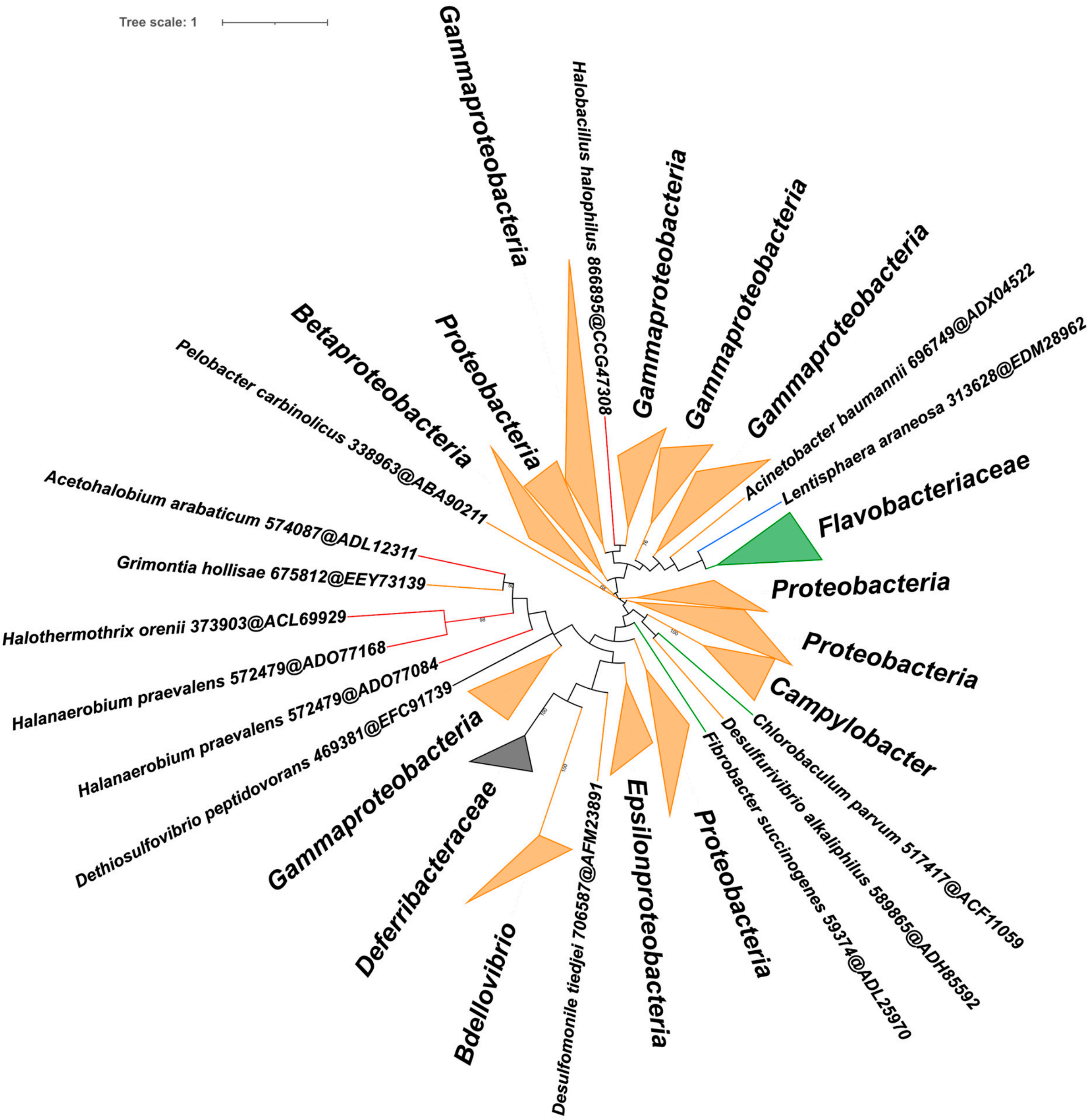

Figure S14: Unrooted TolA tree inferred using RAxML under the LG+F+Γ model. Color key for branches (collapsed included) is: red = Terrabacteria, orange = Proteobacteria, green = FBC superphylum, blue = PVC superphylum, black = other.

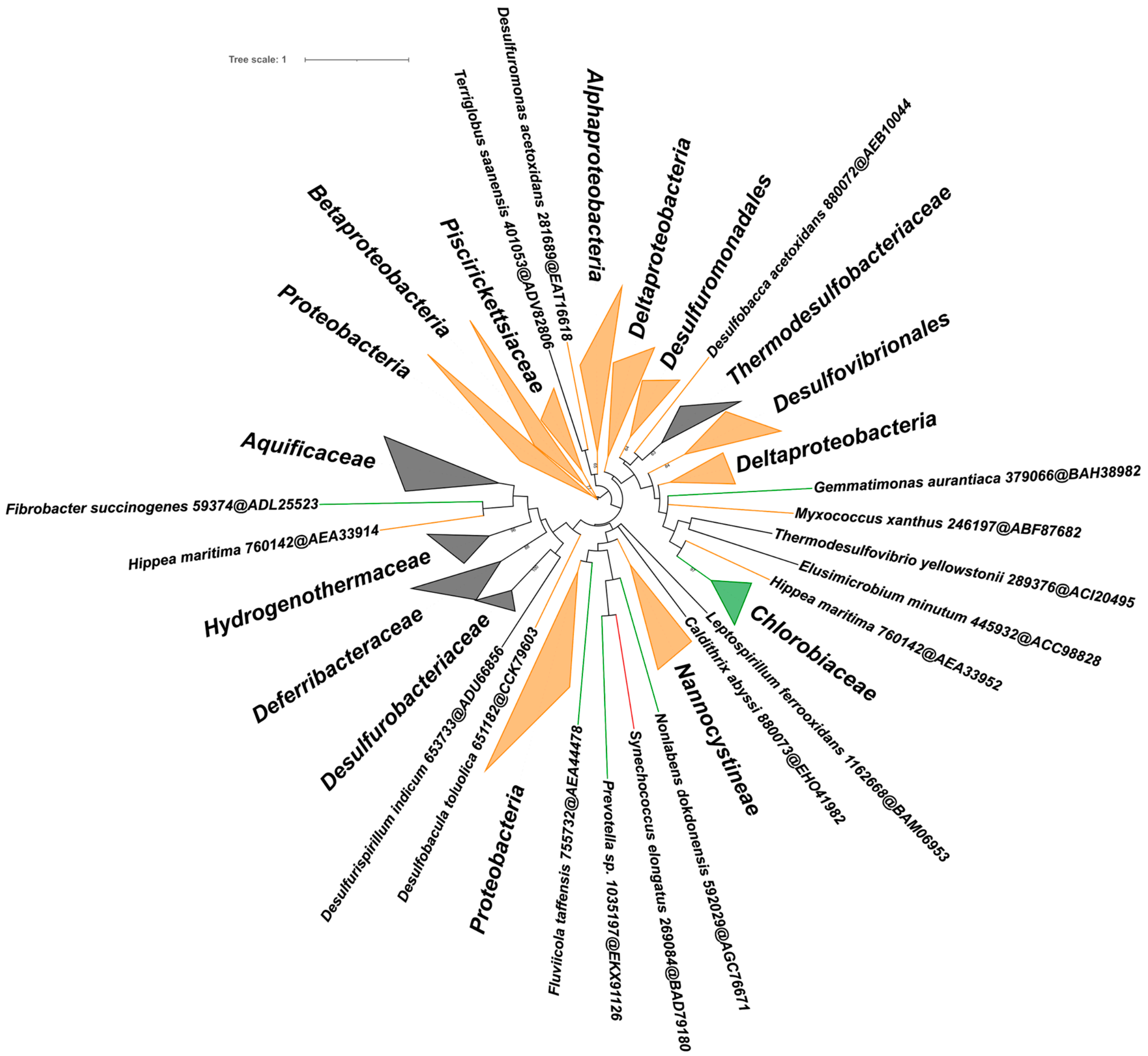

Figure S15: Unrooted YbgF tree inferred using RAxML under the LG+F+Γ model. Color key for branches (collapsed included) is: red = Terrabacteria, orange = Proteobacteria, green = FBC superphylum, blue = PVC superphylum, black = other.

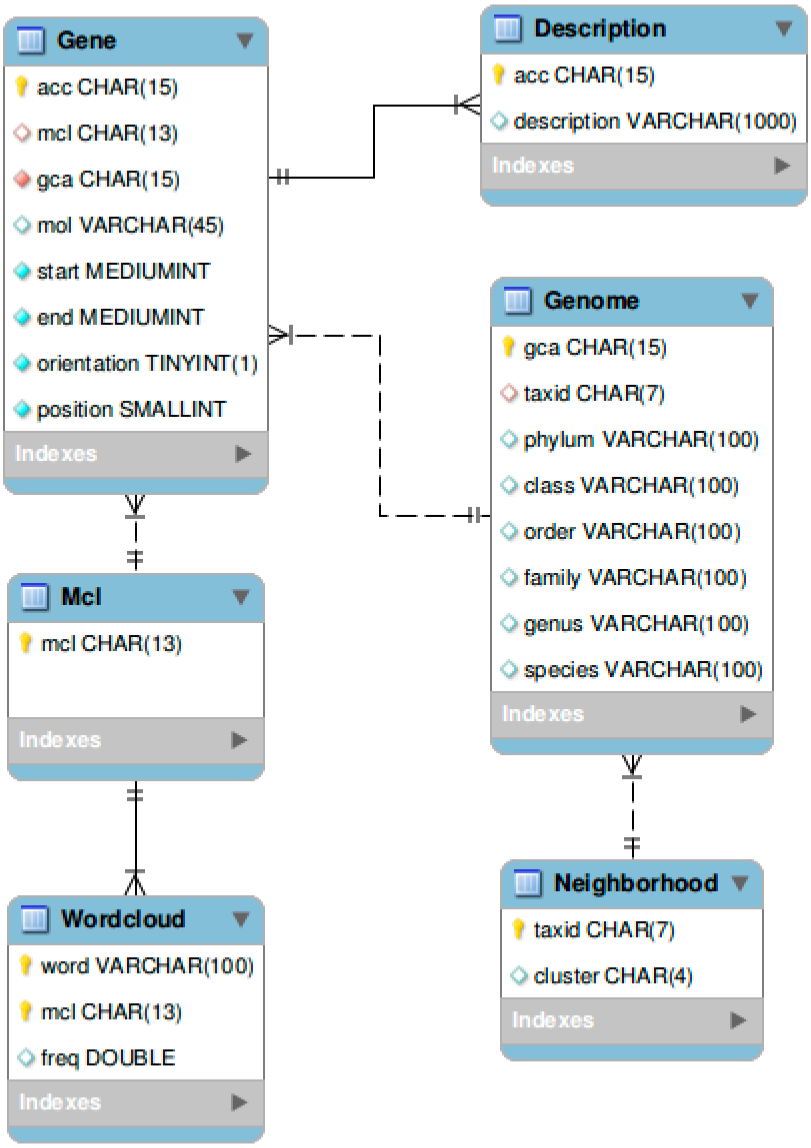

Figure S16: Schema of the MySQL database used by the synteny tool.
